# A Pre-Kidney Transplant Blood-Based Next-Generation Sequencing Assay to Predict Early Acute Rejection

**DOI:** 10.34067/KID.0000001208

**Published:** 2026-05-14

**Authors:** Beatrice P. Concepcion, Oriol Bestard, Milagros Samaniego-Picota, Rohini Prashar, M. Javeed Ansari, Giorgia Comai, Elisa Gessaroli, Lionel Rostaing, Federico Alberici, Roslyn B. Mannon, Joshua Augustine, Alvin Wee, Emilio Poggio, Pierpaolo DiCocco, Nicholae Leca, Saed Shawar, Chandra Bhati, Daniel Maluf, David M. Rothstein, Aravind Cherukuri, Anthony Chang, Fadi Salem, Michael W. Kattan, Jesse D. Schold, Patricia Connolly, Angela Rose, Nehal Doshi, Lorenzo Gallon, Michael J. Donovan

**Affiliations:** 1 Department of Internal Medicine, University of Chicago, Chicago, Illinois; 2 Hospital Universitari Vall d’Hebron, Barcelona, Spain; 3 Henry Ford Health System, Detroit, Michigan; 4 Department of Internal Medicine, Northwestern University, Evanston, Illinois; 5 Nephrology, Dialysis and Kidney Transplant Unit, IRCCS Azienda Ospedaliero-Universitaria di Bologna, Bologna, Italy; 6 CHU Grenoble Alpes, Grenoble, France; 7 ASST degli Spedali Civili di Brescia, Brescia, Italy; 8 University of Nebraska Medical Center, Omaha, Nebraska; 9 Cleveland Clinic, Cleveland, Ohio; 10 Department of Internal Medicine, University of Illinois at Chicago, Chicago, Illinois; 11 Department of Internal Medicine, University of Washington, Seattle, Washington; 12 Department of Kidney Transplant Surgery, Vanderbilt University, Nashville, Tennessee; 13 Department of Internal Medicine, University of Maryland, Baltimore, Maryland; 14 University of Pittsburgh Medical Center, Pittsburgh, Pennsylvania; 15 Mayo Clinic, Jacksonville, Florida; 16 Department of Surgery, University of Colorado Anschutz Medical Campus, Aurora, Colorado; 17 Department of Epidemiology, School of Public Health, University of Colorado Anschutz Medical Campus, Aurora, Colorado; 18 Verici Dx, Franklin, Tennessee; 19 Icahn School of Medicine at Mount Sinai, New York, New York

**Keywords:** acute rejection

## Abstract

**Key Points:**

- Pretransplant early acute rejection risk stratification offers a novel approach to personalized immunosuppression management in patients who received a kidney transplant.
- Stratifying immunologic risk independent of donor characteristics may help guide immunosuppression management in patients who received a kidney transplant.
- The pretransplant risk assessment gene set reflects metabolic and immune pathways involved in T-cell– and antibody-mediated mechanisms of transplant rejection.

**Background:**

Although donor and recipient characteristics are used to estimate graft failure risk, they remain limited in predicting early acute rejection (EAR). We developed and validated a next-generation sequencing assay targeting the pretransplant immunologic profile to predict EAR following kidney transplantation.

**Methods:**

This prospective, international study enrolled 321 kidney transplant participants across 13 sites, forming discovery and validation cohorts. Peripheral blood was collected before transplant and at 1, 3, 6, 12, and 24 months post-transplant, with protocol biopsies performed at 3 and 12 months, as well as any indication biopsies. Biopsies were assessed by a blinded central pathologist according to the 2019 Banff criteria. The pretransplant risk assessment (PTRA) test to evaluate RNA expression of a 29-gene signature algorithm designed to predict EAR risk was clinically validated using 122 deceased donor kidney transplant recipients.

**Results:**

PTRA classified 31 of 122 participants (25%) as high risk and 91 (75%) as low risk. There were nine EAR events in the first 60 days post-transplant: 6/31 (19%) in the high-risk group and 3/91 (3%) in the low-risk group. PTRA discrimination for EAR at 60 days post-transplant yielded an area under the curve of 0.78 (95% confidence interval [CI], 64.4 to 91.5), *P* < 0.001. A cutoff of ≤45 was defined as low risk and >45 as high risk for EAR within 60 days post-transplant. Sensitivity was 0.67 (95% CI, 0.36 to 0.97), specificity 0.78 (95% CI, 0.70 to 0.86), positive predictive value 0.19 (95% CI, 0.05 to 0.33), and negative predictive value 0.97 (95% CI, 0.93 to 1.00). The odds ratio comparing patients identified as high versus low risk by PTRA was 7.04 (95% CI, 1.64 to 30.2), *P* = 0.009.

**Conclusions:**

This study validates the ability of PTRA to stratify kidney transplant recipients as high or low risk for EAR using a pretransplant transcriptomic profile, with implications for graft health and personalized treatment management.

## Visual Abstract

**Figure:**
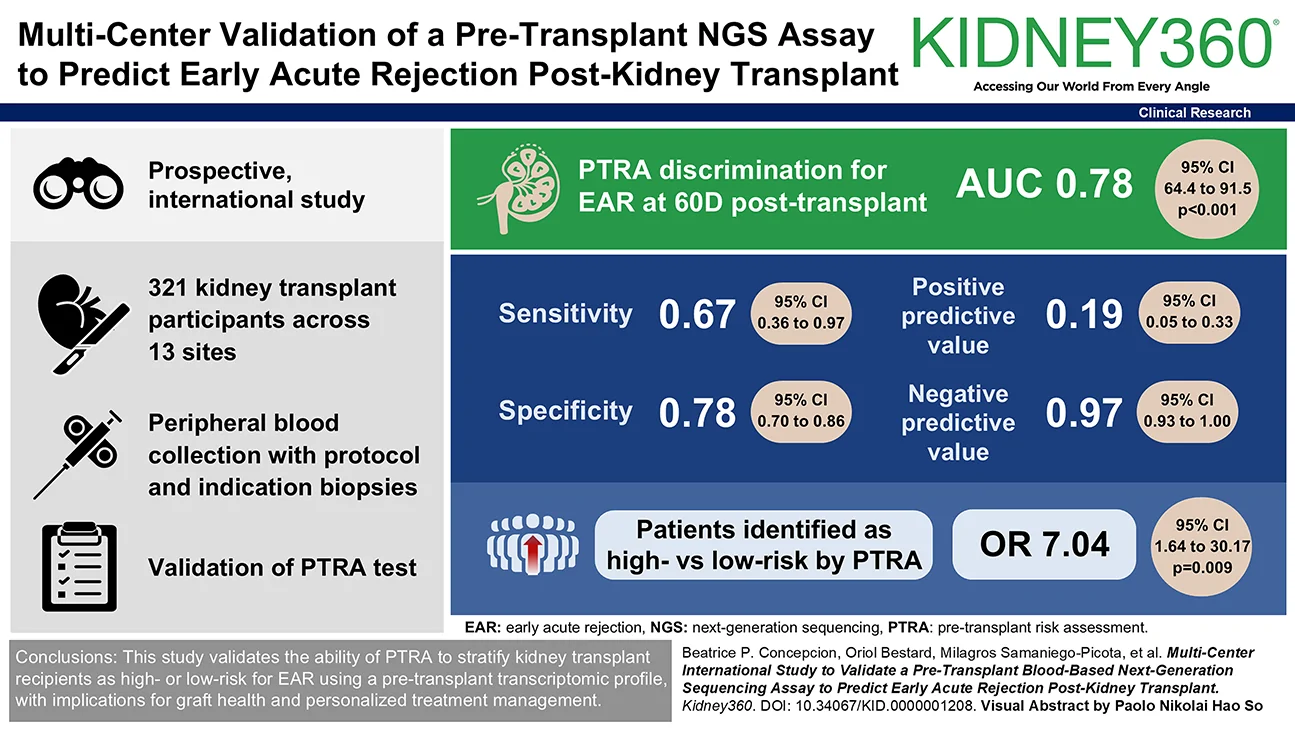

## Introduction

Immunosuppression following kidney transplantation aims to prevent acute rejection (AR) while balancing the risks of toxicity, infection, and malignancy. In recent decades, the incidence of early acute rejection (EAR) has significantly declined, primarily because of the use of more aggressive immunomodulatory regimens.^1^ However, despite the reduction in EAR incidence, long-term graft survival—particularly in recipients of deceased donor (DD) kidneys—has only marginally improved over the past several decades.^1–6^ EAR is an important correlate to graft loss and poor long-term function, emphasizing the importance of early identification and prevention of EAR.^7–10^

In clinical practice, immunosuppressive therapy is typically guided by broad clinical criteria associated with risk, including anti-human leukocyte antigen (HLA) antibodies, race, prior transplantations, and recipient age.^11^ This empiric approach may be insufficiently precise and may expose patients unnecessarily to immunosuppression-related toxicity, infection, and malignancy.

Evidence exists that both the phenotype^6,12,13^ and function^14,15^ of the immune system before transplantation influence the likelihood of AR after transplantation. However, no biomarker has been validated to quantify this risk. A more individualized approach to immunosuppression remains elusive, although ongoing research continues to seek tools that enable more patient-level risk stratification.^16^

Blood-based biomarker testing during the pretransplant phase could improve immune risk stratification by identifying systemic alloimmune perturbation in peripheral blood. Such testing may help differentiate between patients at a higher versus lower risk of post-transplant rejection. Stratifying allograft recipients using pretransplant RNA expression profiles offers the potential for unbiased immunologic phenotyping, which could inform more effective titration of immunosuppressive therapy.^16^

Although HLA matching and the assessment of calculated panel reactive antibodies (cPRA) are accepted methods of immunologic risk assessment, they do not directly measure pretransplant antidonor cellular immunity or the presence of donor-specific antibodies.^6,17^

RNA signature assays represent an innovative step forward, offering more sensitive and specific insights into individual patient risk. Multiomic approaches, including transcriptomics, have been explored to improve the prediction of transplant outcomes. For example, an RNA-based assay performed on preimplant kidney allograft biopsies has been developed to predict long-term graft outcomes.^18^ A urine-based protein signature from DDs has been shown to predict kidney allograft injury response and repair.^19^ In heart transplant recipients, pretransplant recipient blood RNA profiles have been associated with post-transplant mortality.^20^ In addition, *in vitro*-stimulated IFN-*γ* production by pretransplant recipient peripheral blood mononuclear cells has been shown to predict the risk of EAR and may help guide immunosuppression strategies.^21,22^ Finally, a pre-kidney transplant whole-blood RNA sequencing signature has been used to predict EAR post-transplant.^23^

Herin, we report findings from a prospective, multicenter, international trial validating a newly developed 29-gene RNA expression signature, pretransplant risk assessment (PTRA), designed to predict EAR in the first 2 months following kidney transplantation in DD recipients.

## Methods

### Study Participants and Specimens

This study is a prospective, international clinical trial (NCT04727788) designed to validate the Verici Dx transcriptomic test for predicting the risk of AR and chronic allograft damage in kidney transplant recipients. The study was approved by the Advarra Institutional Review Board (PR0049177), and all 13 participating sites adhered to the Declaration of Helsinki. Details regarding trial conduct and specimen handling have been published previously.^24^

From March 2021 to January 2023, 321 participants were enrolled and identified as part of an ongoing 533-participant study. All 321 participants were included on the basis of the availability of pretransplant peripheral blood RNA that met laboratory quality control criteria and access to post-transplant allograft biopsies with adequate material for central pathology assessment. There were 113 patients who appeared in both the pretransplant cohort (*n*=321) and the post-transplant cohort (*n*=151). An additional 37 patients overlapped in the corresponding validation cohort.^24^ One hundred twenty-three (both living donor [LD; *n*=43] and DD [*n*=80] kidney recipients) of the 321 were used for gene discovery and algorithm selection. The combination of LD and DD transplant patients in the training set was initially designed to identify a robust, stable, and biologically diverse gene expression pretransplant immunologic risk profile reflective of the innate metabolic, cellular, and cytokine-mediated processes underlying quiescence, recovery, and rejection in the transplanted kidney regardless of the transplant kidney type. However, in developing the signature, we considered the recipients' immunologic response in the context of established differences between DD and LD kidneys, including the hypoxic state, proinflammatory markers, rejection promoting adhesion molecules, macrophage infiltration, and higher level of injury biomarkers. This resulted in the need to focus on developing a signature from only the 80 DD transplant kidney patients for subsequent validation. Therefore, pretransplant blood from LD and DD transplant recipients was used to discover the gene set, and the DD group was used to train the algorithm. An independent test set comprised 122 DD participants from 11 sites was used for PTRA assay validation. Seventy-six LD participants were sequestered for future studies.

**Figure 1:**
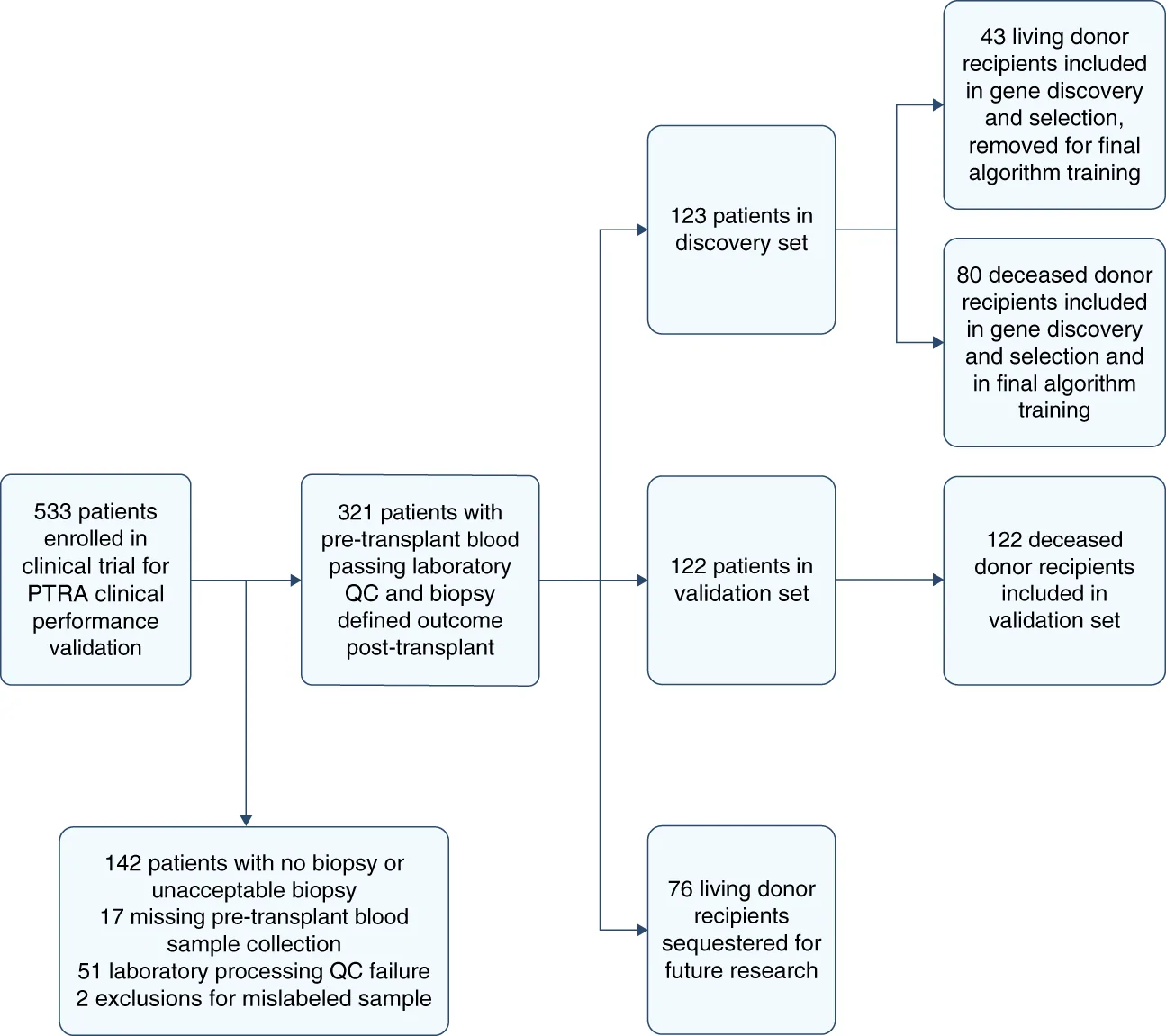
**CONSORT diagram.** CONSORT, Consolidated Standards of Reporting Trials; PTRA, pretransplant risk assessment; QC, quality control.

Numerous studies have reported that LD transplants have lower rejection rates compared with DD transplants.^25,26^ This difference persists even when DDs are stratified by donor type (brain death versus cardiocirculatory death donors),^26^ which guided the focus on the DD population here. The study Consolidated Standards of Reporting Trials diagram is provided in Figure 1. This observational study adhered to the Strengthening the Reporting of Observational Studies in Epidemiology reporting guidelines.

After transplantation, participants were followed at 1, 3, 6, 12, and 24 months for specimen collection and clinical updates. In the validation cohort, protocol kidney biopsies were performed at the 3- and 12-month visits in 99 and 79 participants, respectively. Indication biopsies (*n*=60) were performed in 45 of the 122 validation cohort participants between 6 days and 591 days post-transplant, depending on site-specific practices.

The intended use of PTRA was to apply a pretransplant peripheral blood gene signature to predict the risk of EAR within 2 months post-transplant. Blood samples were collected within 2 weeks before transplant for all patients in the validation cohort, except one whose transplant was canceled, and then, the patient received a transplant 7 weeks later.

All kidney biopsies were first evaluated by local pathologists using hematoxylin and eosin, special stains, and C4d immunofluorescence/immunohistochemistry. Biopsies were then reviewed by a central nephropathologist who was blinded to local reports and provided primarily digital slides and limited clinical data. The 2019 Banff criteria were used to assess *T*-cell– and antibody-mediated rejection. Chronic damage, such as interstitial fibrosis and tubular atrophy with or without inflammation, was assessed using the Chronic Allograft Damage Index and Banff lesion scores. In cases of diagnostic discrepancy, a third pathologist adjudicated the case using all available images and data.

### Isolation and Analysis of RNA for the PTRA Signature Gene Assay

Total RNA was isolated from the peripheral blood of the 123 LD and DD recipients in the PTRA discovery set and from the 122 DD recipients in the validation cohort using the Promega Maxwell simplyRNA kit. cDNA libraries were generated using the Illumina Stranded mRNA Library Prep Ligation Kit and sequenced on an Illumina NextSeq 2000 platform.

### Differential Expression and Feature Selection

A more detailed description of gene selection is provided in Figure 1 and Supplemental Methods. Briefly, good quality sequencing reads were trimmed and prefiltered before downstream processing to ensure reliable gene-level quantification. Differentially expressed genes from this set were identified using a combined statistical approach incorporating DESeq2, limma-voom, and edgeR. Feature selection was guided by optimization of the area under the curve (AUC) in cross-validation, with SDA providing regularized projections to stabilize selection in the high-dimensional RNA-seq setting. A logistic regression model was developed using hyperparameter tuning and regularization. Biologic pathway and functional analyses were performed on the finalized PTRA gene set to support biologic interpretation of the signature, which are summarized in Supplemental Table 1. These analyses were conducted after model development and validation and were not used for feature selection or model optimization. Functional annotations of PTRA genes were performed using curated biologic knowledge bases to identify shared immune, metabolic, and inflammatory processes represented within the signature. Pathway enrichment was additionally assessed using gene set enrichment analysis, with enrichment significance evaluated using *P* values.

**Figure 2:**
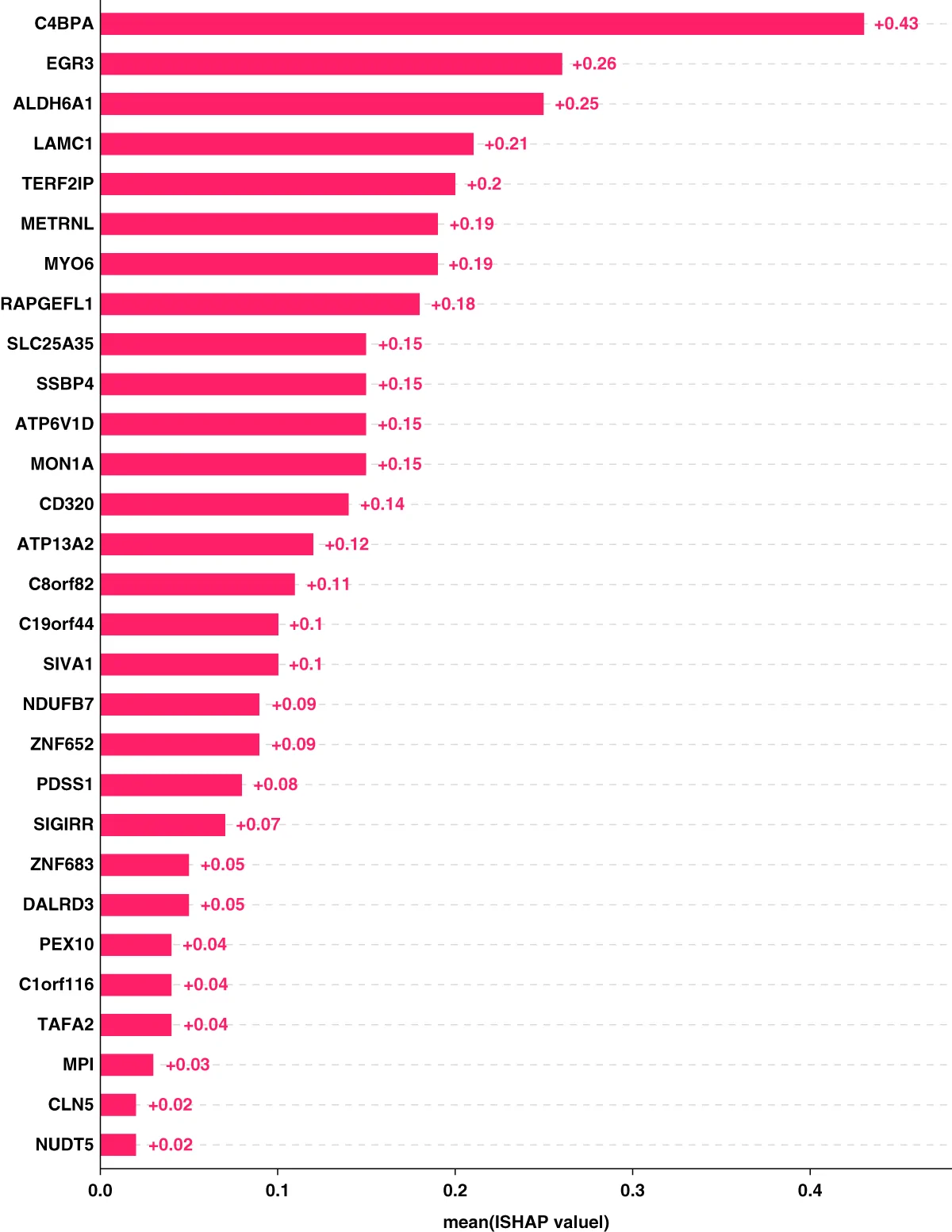
**Twenty-nine genes in the PTRA signature used to generate a predictive risk score for EAR.** This SHAP plot illustrates the degree of importance (highest to lowest) of the 29 genes in the PTRA signature used to generate a prediction risk score for EAR. EAR, early acute rejection; SHAP, SHapley Additive exPlanations.

**Figure 3:**
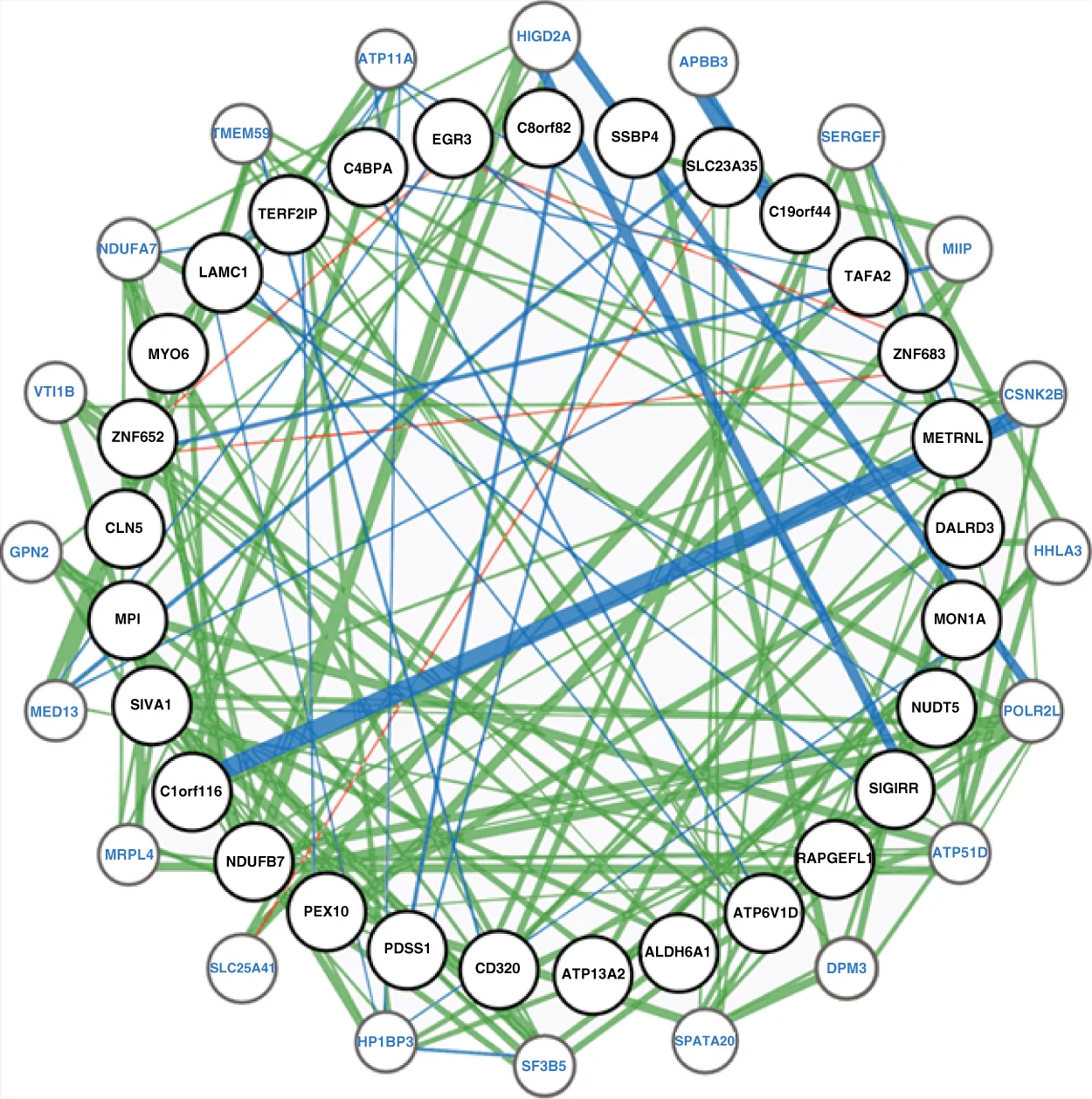
Assessment of the 29 genes interrelatedness of PTRA gene signature.

The resulting model comprised a final select set of 29 genes associated with prediction of EAR. Each gene contributed collectively and differentially to the PTRA algorithm for risk prediction (Figure 2). A complete list of the 29 genes, including names, Ensembl IDs, functional roles, and supporting references, is provided in Supplemental Table 1. Gene-based biologic and pathway analyses were performed on the final signature genes, including assessments of gene–gene relationships, which were further examined through assessment of coexpression patterns, reported protein–protein interactions, and shared functional domains to evaluate biologic connectivity within the signature. The results from this analysis were used to contextualize the biologic relevance of the PTRA gene set. In addition, literature references associated with each gene were reviewed to verify reported functional roles and previously described associations with immune regulation, inflammation, and transplant-related outcomes (Figure 3). The PTRA score was derived from a prespecified, locked logistic regression classifier trained on the finalized 29-gene signature. For each sample, the model generated a predicted probability of EAR on the basis of normalized gene expression values and fixed model coefficients. Predicted probabilities were transformed by linear scaling (multiplication by 100) to produce a standardized score ranging from 0 to 100, with higher scores indicating greater predicted risk. A predefined threshold was applied to the standardized score to classify patients into low- and high-risk groups. All parameters and thresholds were locked before validation and were not modified during testing.

### Model Development and Validation

The final model consisted of a 29-gene signature with logistic regression classifier algorithm. Importantly, all filtering, feature elimination, and model comparisons were restricted to the training data only; the independent test cohort remained sequestered until final evaluation. This design prevents information leakage and ensures that model performance reflects generalizability rather than overfitting.

### Primary Objective

The primary objective was to validate the prognostic performance of PTRA in predicting low or high risk of EAR by calculating the time-dependent area under the receiver operating characteristic (AUROC) curve at 60 days and correlate with histopathologic outcomes from kidney biopsies defined by the 2019 Banff criteria.^27^ Patients were divided into demographic and event balanced training and validation cohorts, and samples from DD recipients were separated from LD recipients. With 122 patients available for analysis, and a 30% expected prevalence, this study was predetermined to have an 80% power to detect an AUROC curve of at least 0.66.

### Exploratory Analysis of Immune Cell Type Populations

We performed exploratory immune profiling using iSort Fractions, an artificial intelligence-powered digital cytometry platform that deconvolutes bulk RNA sequencing data to estimate relative proportions of immune cell subsets on the basis of reference signature matrices.^28^ iSort Fractions was applied to gene expression data from the 321 patients with pretransplant blood consisting of 202 DD and 119 LD recipients with biopsy-defined post-transplant outcomes to estimate immune cell composition. We further compared pretransplant immune profiles between patients who experienced EAR versus those who did not. Differences in immune cell populations between DD and LD recipients were also evaluated.

### Statistical Analysis

Baseline demographic and clinical characteristics of PTRA study participants with and without rejection were compared using the Wilcoxon rank-sum test for continuous variables and the chi-squared test for categorical variables. The PTRA model was applied to pretransplant blood from DD kidney recipients to predict risk of EAR within 60 days post-transplant. Competing risks including borderline rejection, graft loss, or death within 60 days, were classified as nonevents unless preceded by biopsy-proven AR. Patients without a biopsy or biopsy-confirmed EAR within 60 days were also considered nonevents.

We calculated the AUROC for the defined time-to-event within 60 days. In the absence of a prespecified clinical benchmark, the primary analysis compared the AUC of PTRA with an AUC of 0.5. Additional performance metrics included positive predictive value, negative predictive value, sensitivity, specificity, and hazard ratio. In addition, we calculated AUROC using both class 1 and class 2 panel reactive antibody (PRA) levels for comparison. For these analyses, we evaluated the predicative value for biopsy-proven AR using high class 1 and class 2 PRA above 20%, as well as comparing the predictive values considering both as continuous measures of risk.

Comparisons between groups were performed using two-way ANOVA. In cases with only two groups, two-way ANOVA is equivalent to a two-way *t* test. All statistical analyses were conducted using SAS 9.4. Immune cell proportions from iSort were min/max normalized within cell types and compared across groups using two-way ANOVA (ggpubr package in *R*).

## Results

### Signature Gene Selection and Function

The three genes with the greatest network interaction, based on correlational coefficients, are described in Table 1. The interconnected network of the 29 genes comprising the PTRA signature is represented in a modified Circos plot in Figure 3. As depicted, all 29 genes exhibited a high degree of coexpression, with a modest but variable level of interaction between select genes within the extended gene network. Specifically, the inner ring represents the 29 signature genes, whereas the outer ring contains biologically related pathway network genes. Gene interactions are depicted in blue, coexpression in green, and shared protein domains in red. The thickness of each line reflects the strength of the interaction.

**Table 1:** Top three pretransplant risk assessment genes with the greatest network interaction

Signature Gene	Interacting Gene	Correlation Coefficient	Role
C1orf116	CSNK2B	0.049	C1orf116 is a novel driver of epithelial phenotype in epithelial-to-mesenchymal transition. CSNK2B encodes the regulatory beta subunit of CK2, a protein kinase that regulates many cellular processes including metabolic pathways, signal transduction, transcription, translation, and replication
C19orf44	APBB3	0.041	C19orf44 is postulated to play a role in memory B cells. APBB3 protein binding is predicted to be involved in negative regulation of cell cycle phase transitions; negative regulation of transcription by RNA polymerase II; and positive regulation of protein secretion. It is also predicted to act upstream of or within the negative regulation of the apoptotic process
SIGIRR	HIGD2A	0.023	SIGIRR dampens the signaling pathway leading to NF-kB activation. In *T* cells and epithelial cells, TIR8/SIGIRR inhibits IL-1–dependent activation of the Akt-mTOR pathway and JNK, thus controlling cell proliferation and survival. *In vivo* studies demonstrate that TIR8/SIGIRR acts as a nonredundant negative regulator in various inflammatory conditions mediated by ILRs or TLRs In models of renal ischemia/reperfusion or kidney transplantation, TIR8/SIGIRR-deficient mice showed increased renal injury or severe graft rejection, respectively. These outcomes were associated with excessive cytokine and chemokine production, leading to enhanced leukocyte recruitment and an amplified adaptive immune response against donor antigens HIGD2A is a subunit of the cytochrome c oxidase complex, the terminal enzyme in the mitochondrial respiratory chain. In animal models, it has been shown to enhance cell survival under hypoxic conditions

AKT, protein kinase B; APBB3, amyloid beta precursor protein binding family B member 3; C19orf44, chromosome 19 open reading frame 44; CK2, casein kinase 2; CSNK2B, casein kinase 2 *β*; C1orf116, chromosome 1 open reading frame 116; HIGD2A, hypoxia inducible domain family member 2A; IL-1, interleukin-1; mTOR, mammalian target of rapamycin; NF-kB, NF kappa-light-chain-enhancer of activated B cells; PTRA, pretransplant risk assessment; SIGIRR, single Ig and toll-interleukin 1 receptor domain; TIR8, toll interleukin-1 receptor 8; TLR, toll-like receptor.

**Figure 4:**
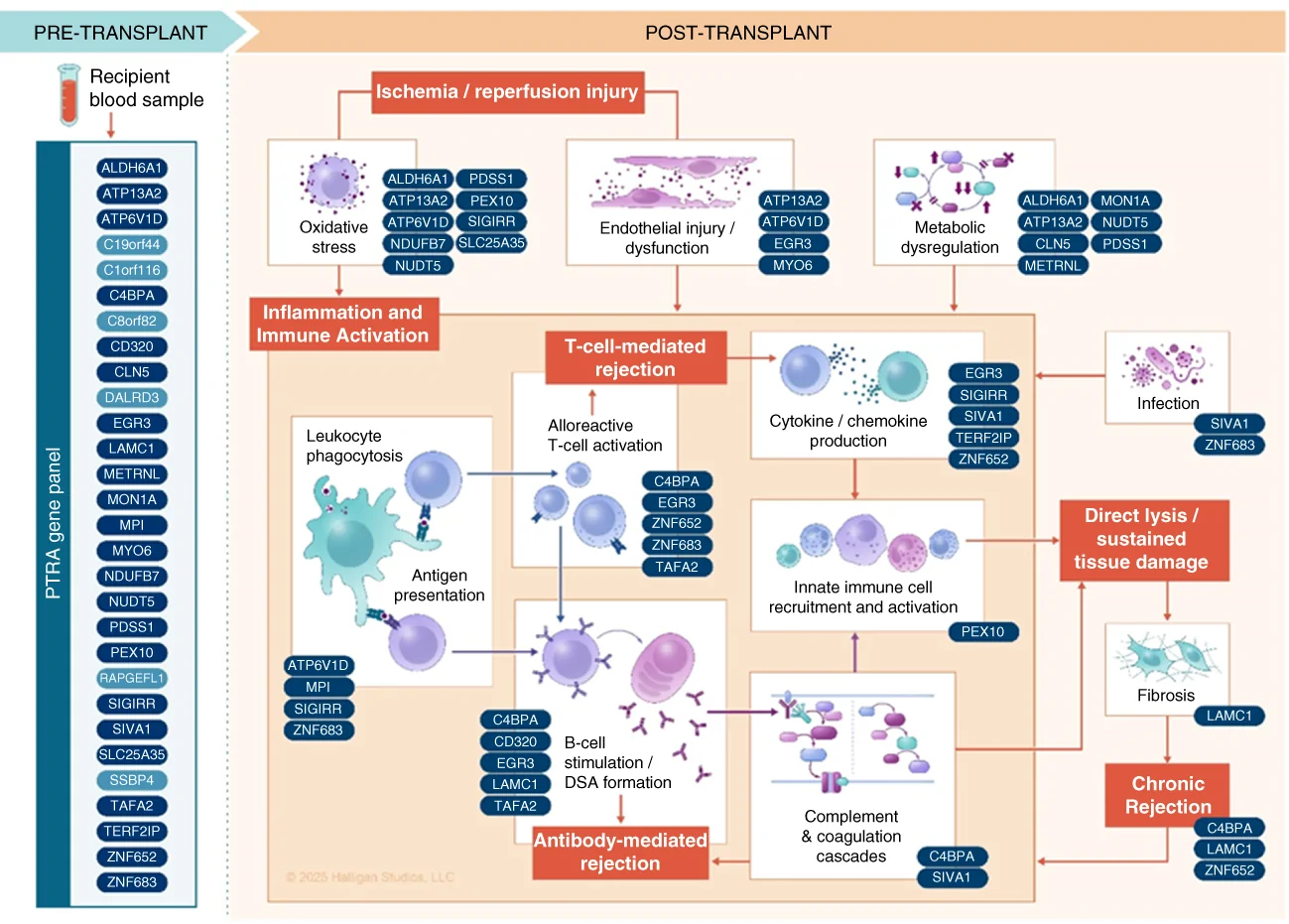
**Biologic pathways of activity in the select set of PTRA signature genes.** DSA, donor-specific antibodies

Notably, the biologic pathways associated with the top ten genes in the PTRA signature were predominantly linked to inflammation and immune activation. As shown in Figure 4, these genes were involved in chemokine and complement initiation, regulatory *T*-cell activation, T-cell signaling, ATP/ADP metabolism, autophagy-lysosomal trafficking, lipid processing, cell migration, and epithelial-mesenchymal transition. The remaining 19 genes were related to RNA processing and immune cell responses, including B-cell proliferation, IgG secretion, cluster of differentiation (CD)8^+^ *T*-cell infiltration, and natural killer (NK) cell activation. Of note, there was no overlap between the 29 gene PTRA signature and the 17 genes in the post-transplant assay.^24^

### Immune Cell Population Analysis

**Figure 5:**
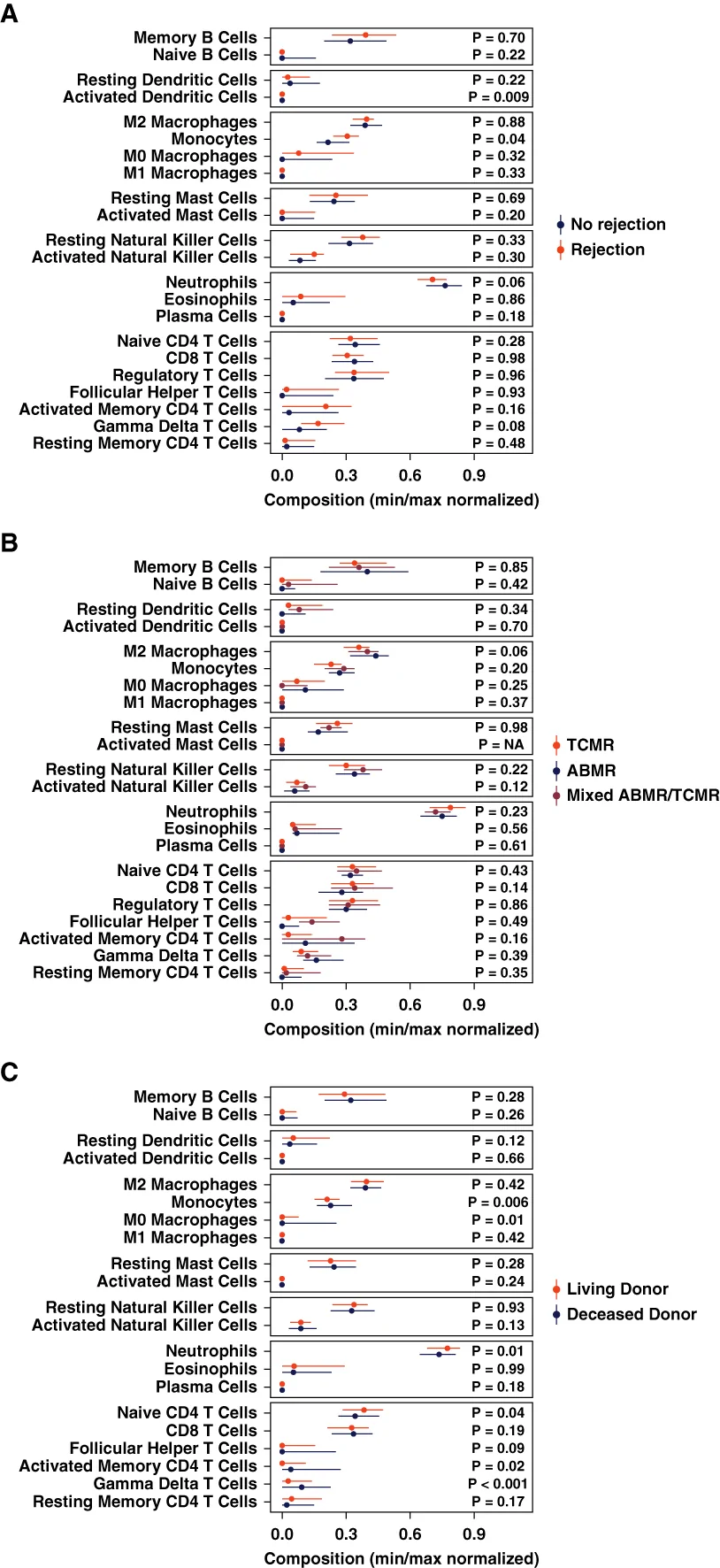
**Immune cell populations in pretransplant blood.** (A) Immune cell populations in pretransplant blood for trial participants who experienced EAR (*n*=22) compared with those who did not (*n*=180). (B) Immune cell populations in pretransplant blood in study patients with EAR stratified by rejection type. (C) Immune cell populations in pretransplant blood for trial participants who received a DD (*n*=202) transplant in comparison with those who received a LD (*n*=119) transplant. ABMR, antibody-mediated rejection; DD, deceased donor; LD, living donor; max, maximum; min, minimum; TCMR, *T*-cell–mediated rejection.

Immune cell type deconvolution was performed on pretransplant blood samples from the 202 DD recipients using RNA sequencing-based estimates of 22 immune cell types. Participants who developed EAR had a statistically higher proportion of monocytes, with elevated levels of NK cells, activated memory CD4^+^ T cells, and *γδ T* cells compared with those without EAR (*P* = NS, Figure 5A).

We next evaluated whether immune cell populations differed by EAR subtype. Although the sample size was limited, patients with acute antibody-mediated rejection had significantly higher levels of macrophage, with similar levels of circulating monocytes. By contrast, cases of mixed rejection showed elevated NK cells, CD4^+^, and CD8^+^ *T* cells, whereas *T*-cell–mediated rejection cases were characterized by increased CD4^+^ and CD8^+^ *T* cells alone (Figure 5B).

Differences in pretransplant immune cell composition were also examined by study participants who received a DD versus a LD donor type. DD transplant recipients (*n*=202) exhibited significantly higher proportions of macrophages, monocytes, regulatory *T* cells, activated memory CD4^+^ T cells, and *γδ T* cells compared with LD recipients (*n*=119; Figure 5C).

### Performance of the PTRA 29-Gene Signature to Predict EAR

Clinical and demographic characteristics of the validation cohort are provided in Table 2. The 122 patients had a median age of 57 years, 54% were male, 66% identified as White, and 30% as Black. Of these participants, 14% had a history of previous failed transplants and 99% were ABO compatible. The median donor age was 55 years, and 100% of donors were DD, including 55% characterized as standard criteria, 27% as expanded criteria, and 18% as donation after cardiac death. There were no site-specific restrictions on immunosuppressive regimens, and all patients received induction therapy (Table 3).

**Table 2:** Validation cohort clinical characteristics

Characteristic	Total (*N*=122)	Low-Risk Score (*n*=91)	High-Risk Score (*n*=31)	*P* Value
**Donor age, yr**				
Mean (SD)	51.4 (17.8)	50.6 (18.9)	53.8 (14.3)	0.31
Median [min–max]	55.5 [2.00–81.0]	54.0 [2.00–81.0]	57.0 [16.0–75.0]	
**Donor sex, *No.* (%)**				
Male	78 (64)	63 (69)	15 (48)	0.06
Female	44 (36)	28 (31)	16 (52)	
**Donor race, *No.* (%)**				
Asian	0 (0)	0 (0)	0 (0)	NA
Black	8 (7)	6 (7)	2 (7)	
Pacific island	0 (0)	0 (0)	0 (0)	
Missing	17 (14)	14 (15)	3 (10)	
White	97 (80)	71 (78)	26 (84)	
**Donor ethnicity, *No.* (%)**				
Hispanic/Latino	4 (3)	3 (3)	1 (3)	1.00
Not Hispanic/Latino	102 (84)	75 (82)	27 (87)	
Missing	16 (13)	13 (14)	3 (10)	
**Donor status, *No.* (%)**				
Living related donor	0 (0)	0 (0)	0 (0)	NA
Living unrelated donor	0 (0)	0 (0)	0 (0)	
Standard criteria donor	67 (55)	51 (56)	16 (52)	
Expanded criteria donor	33 (27)	24 (26)	9 (29)	
Donors after cardiac death	22 (18)	16 (18)	6 (19)	
**Recipient age, yr**				
Mean (SD)	55.1 (13.4)	54.4 (13.8)	57.1 (12.3)	0.32
Median [min–max]	57.0 [24.0–77.0]	57.0 [24.0–77.0]	58.0 [27.0–76.0]	
**Recipient sex, *No.* (%)**				
Male	66 (54)	54 (59)	12 (39)	0.06
Female	56 (46)	37 (41)	19 (61)	
**Recipient race, *No.* (%)**				
American Indian or Alaska native	0 (0)	0 (0)	0 (0)	NA
Asian	3 (3)	1 (1)	2 (7)	
Black	37 (30)	29 (32)	8 (26)	
Missing	2 (2)	1 (1)	1 (3)	
Pacific island	0 (0)	0 (0)	0 (0)	
White	80 (66)	60 (66)	20 (65)	
**Recipient ethnicity, *No.* (%)**				
Hispanic/Latino	6 (5)	4 (4)	2 (7)	0.99
Not Hispanic/Latino	115 (94)	87 (96)	28 (90)	
Missing	1 (1)	0 (0)	1 (3)	
**Recipient medical history, *No.* (%)**				
Diabetes	28 (23)	17 (19)	11 (36)	0.09
Hypertension	95 (78)	71 (78)	24 (77)	1.00
Glomerulonephritis	23 (19)	16 (18)	7 (23)	0.27
Polycystic kidney disease	15 (12)	13 (14)	2 (7)	0.41
Congenital abnormality	2 (2)	2 (2)	0 (0)	0.99
Family history of kidney disease	4 (3)	2 (2)	2 (7)	0.57
IgA nephropathy/Berger disease	7 (6)	7 (8)	0 (0)	0.25
Systemic lupus erythematosus	3 (3)	2 (2)	1 (3)	1.00
Focal segmental glomerulosclerosis	4 (3)	4 (4)	0 (0)	0.55
Other kidney disease	29	18	11	0.53
**Prior kidney transplant, *No.* (%)**				
0	105 (86)	79 (87)	26 (84)	0.72
1	16 (13)	11 (12)	5 (16)	
2	1 (1)	1 (1)	0 (0)	
**ABO incompatibility, *No.* (%)**				
No	121 (99.2)	90 (99)	31 (100)	1.00
Yes	1 (0.8)	1 (1)	0 (0)	
**Cold ischemia time, h**				
Mean (SD)	16.6 (8.78)	16.9 (9.06)	15.9 (8.01)	0.57
Median [min–max]	14.4 [3.00–41.2]	14.3 [3.00–41.2]	14.5 [3.53–35.7]	
Missing	2 (2)	2 (2.2)	0 (0)	
**Administered therapies, *No.* (%)**				
Induction	122 (100)	91 (100)	31 (100)	NA
Desensitization, preformed DSA	3 (3)	3 (3)	0 (0)	NA
*De novo* DSA	7 (6)	5 (6)	2 (6.5)	NA
**PRA class 1**				
Positive	38 (31)	28 (31)	10 (32)	1.00
Negative	68 (56)	50 (55)	18 (58)	
Missing	16 (13)	13 (14)	3 (10)	
**PRA class 1 %**				
<30	80 (66)	58 (64)	22 (71)	0.85
≥30	26 (21)	20 (22)	6 (19)	
Missing	16 (13)	13 (14)	3 (10)	
**PRA class 2**				
Positive	40 (33)	29 (32)	11 (36)	1.00
Negative	66 (54)	49 (54)	17 (55)	
Missing	16 (13)	13 (14)	3 (10)	
**PRA class 2 %**				
<30	71 (58)	54 (59)	17 (55)	0.56
≥30	35 (29)	24 (26)	11 (36)	
Missing	16 (13)	13 (14)	3 (10)	
**HLA mismatch (A, B, DRB, DQB)**				
0–4	44 (36)	30 (33)	14 (45)	0.32
5–8	78 (64)	61 (67)	17 (55)	

DSA, donor-specific antibodies; HLA, human leukocyte antigen; Max, maximum; Min, minimum; NA, not applicable; PRA, panel-reactive antibody.

**Table 3:** Induction therapy regimes

Induction Regimen, *No.* (%)	Total (*N*=122)	Low-Risk Score (*n*=91)	High-Risk Score (*n*=31)
ATG/thymoglobulin+steroid	82 (67)	63 (69)	19 (61)
IL2RA+steroid	25 (21)	19 (21)	6 (19)
Campath 1H+steroid	12 (10)	9 (10)	3 (10)
ATG/thymoglobulin and IL2RA+steroid	3 (3)	0 (0)	3 (10)

ATG, antithymocyte globulin; IL2RA, IL-2 receptor subunit *α*.

**Figure 6:**
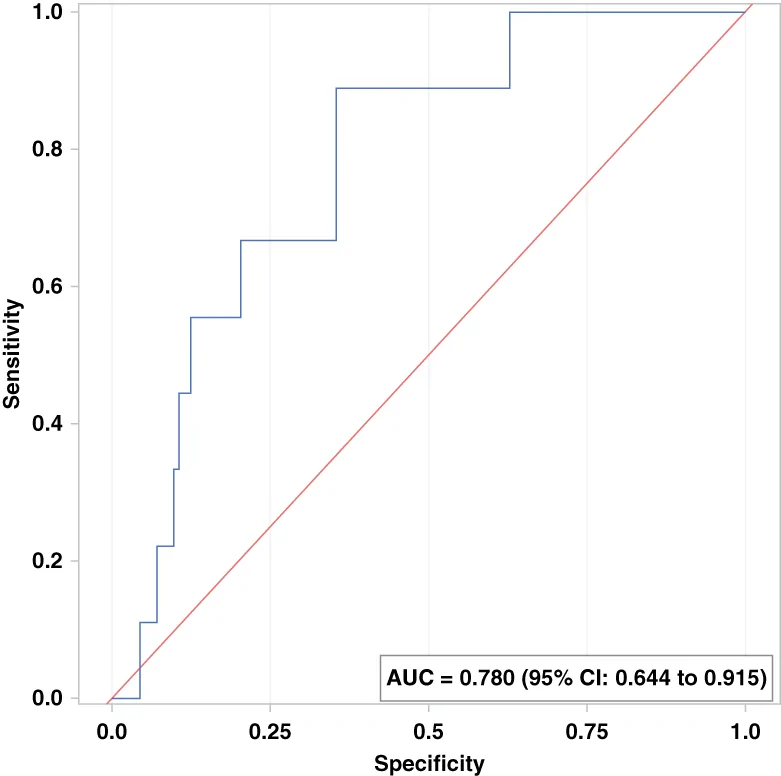
**ROC curve of clinical performance of the PTRA.** AUC, area under the curve; CI, confidence interval; ROC, receiving operating characteristic.

The 29-gene PTRA assay was evaluated in the validation cohort using receiver operating characteristic curve analysis for rejection within 60 days post-transplant. The AUROC was 0.78 (95% confidence interval [CI], 0.64 to 0.92), compared with the baseline clinical model AUROC of 0.50 (95% CI, 50 to 50; *P* < 0.001, Figure 6). HLA mismatches (A, B, DRB, and DQB) and cPRA levels ≥30% (class 1 or class 2) were analyzed using a competing risks regression model; neither of which showed a significant association with EAR (*P* = 0.9 and *P* = 0.4, respectively). The AUROC for both class 1 and class 2 PRA treated both as dichotomous risk levels or as a continuous basis were all <0.6 (Supplemental Figure 2). Moreover, there was no correlation between PTRA results and dialysis duration (r=−0.03, *P* = 0.74), contrasting with previous findings that linked pretransplant IFN-*γ* induction with EAR risk.^21^

Using a model-derived threshold of 45 on the 0–100 scale, the 122-patient validation cohort was stratified into low-risk (≤45) and high-risk (>45) groups. The threshold was determined by using the optimized sensitivity and specificity of the assay with respect to the correct identification of a rejection event. This classification identified 31 patients (25%) as high risk and 91 (75%) as low risk. As noted in Table 2, traditional clinical risk factors—including recipient age, sex, race, underlying disease, history of failed transplant, and sensitization—did not significantly differ between the high- and low-risk PTRA groups. The number of HLA mismatches, dialysis duration, and the use of induction therapy were also similar between groups. Performance metrics for the model are provided in Table 4. The high odds ratio of 7.04 (95% CI, 1.64 to 31.2; *P* = 0.009) between PTRA high- and low-risk groups underscores the statistically significant association of the PTRA score with EAR risk.

**Table 4:** Clinical validation of pretransplant risk assessment performance

Validation Model	Total (*N*=122)	*P* Value
>45, low-risk, *No.* (%)	91 (75)	
≤45, high-risk, *No.* (%)	31 (25)	

Validation performance metric	Performance (95% CI)	
Sensitivity	0.67 (0.36 to 0.97)	
Specificity	0.78 (0.70 to 0.86)	
OR	7.04 (1.64 to 31.2)	*P* = 0.009
PPV	19 (0.05 to 0.33)	
NPV	97 (0.93 to 1.00)	
AUROC	0.78 (65.4 to 91.6)	*P* < 0.001

AUROC, area under the receiver operator characteristic curve; CI, confidence interval; NPV, negative predictive value; OR, odds ratio; PPV, positive predictive value.

All 122 participants in the validation cohort had biopsy-defined outcomes. Within 60 days post-transplant, 18 biopsies were performed, all classified as for-cause. Among these, nine (50%) showed rejection, and five (28%) showed no rejection. An additional four (22%) were classified as borderline rejection and were considered competing risks and nonevents for the primary analysis (Table 5). Beyond 60 days, 28 participants experienced AR, and eight had borderline rejection. Graft loss and death were assessed at 24 months, and PTRA risk scores were not associated with 2-year outcomes.

**Table 5:** Biopsy-defined outcomes 24 months post-transplant

*No.* (%)	≤60D (*n*=18)	>60D (*n*=104)
**For cause**		
Borderline	4 (22)	1 (1)
TCMR IA or higher	1 (6)	3 (3)
ABMR	4 (22)	2 (2)
Mixed	4 (22)	2 (2)
No rejection	5 (28)	4 (4)
**Surveillance**		
Borderline	NA	7 (7)
TCMR IA or higher	NA	9 (9)
ABMR	NA	9 (9)
Mixed	NA	3 (3)
No rejection	NA	64 (62)

ABMR, acute antibody-mediated rejection; D, days; NA, not applicable; TCMR, *T*-cell–mediated rejection.

## Discussion

In this study, we identified a 29-gene transcriptomic signature in pretransplant peripheral blood that predicts EAR in DD kidney transplant recipients. Previous studies have shown that blood-based gene signatures can stratify immunologic risk in transplant recipients and mirror gene expression patterns used to diagnose or predict disease in autoimmune conditions^29,30^ and cancer.^31^ In contrast to earlier work,^23^ our transcriptomic analysis revealed enrichment of CD4^+^ activated memory *T* cells, *γδ T* cells, NK cells, and migrating CD8^+^ *T* cells in patients at a higher risk of EAR—accompanied by increased activation of *T*-cell receptor signaling pathways.

Although clinical variables such as recipient age, race, prior transplantation, and sensitization have been associated with rejection risk,^23^ none of the measured clinical parameters independently predicted EAR in our study. Previous studies have shown that total CD8^+^ *T*-cell counts alone are not predictive of EAR,^32,33^ supporting our approach of combining gene expression with immune cell type and pathway-level analyses. NK cells continue to emerge as relevant players in allograft rejection through mechanisms such as lysis of target cells lacking HLA class 1 expression and antibody-dependent cellular cytotoxicity.^34,35^

Currently, most kidney transplant recipients receive standardized immunosuppressive regimens, with more aggressive induction therapy often reserved for patients perceived as high risk, typically based on broad criteria such as high cPRA levels. However, these conventional risk metrics lack precision, which can lead to over-immunosuppression and its attendant risks, including toxicity, infections, and malignancies.^36^ The ability to intensify immunosuppression in truly high-risk individuals while safely minimizing therapy in low-risk patients represents a key unmet need in transplant medicine and may reduce post-transplant morbidity and mortality.

Our findings show that pretransplant peripheral blood gene signatures can reflect an individual's immune profile and prognosticate EAR with high discriminatory performance. This aligns with emerging data on shared transcriptomic pathways across organ types involved in rejection.^14^ In that work, universal rejection-related signaling—spanning heart, lung, liver, and kidney—was identified, reinforcing the concept that RNA profiles can offer patient-specific insight into allograft biology. In our cohort, the 29-gene PTRA signature captured immune activity consistent with known rejection pathways, including chemokine and cytokine signaling, *T*-cell activation and regulation, and early components of antibody-mediated injury.

Importantly, the predictive value of PTRA was mostly independent of donor characteristics, such as age, race, repeat transplant, and cPRA (with the notable exception of DD versus LD), suggesting an intrinsic recipient-specific inflammatory and rejection phenotype. The intercorrelation of the biologic functions of the 29 genes was further supported by differences in immune cell-type proportions observed between patients who experienced EAR and those who did not (Figure 3). Thus, the ability of PTRA next-generation sequencing to capture an inflammatory phenotype has the potential to identify patients with the greatest need for increased immunosuppression to prevent rejection. As such, we observed clear differences in immune cell composition between DD and LD recipients, suggesting that pretransplant molecular signals may vary meaningfully on the basis of donor type and could influence risk assessment (Figure 5C).

Our data also support the clinical utility of a low-risk PTRA score, which identified individuals with a very low likelihood of developing EAR. This opens the possibility of safely minimizing immunosuppressive therapy in these patients—reducing exposure to toxic agents without compromising graft outcomes. Side effects from lifelong immunosuppression are nearly universal and cause a substantial daily burden to our patients.^37^ Current regimens have been largely unchanged for 15 years, and a one-size-fits-all approach has permeated the transplant field. There is an urgent need for safer immunosuppressive minimization that improves long-term quality of life without increasing rejection risk and, at the same time, avoids infections and cancers associated with overimmunosuppression. As a continuous predictor of EAR within 60 days post-transplant, the PTRA assay has the potential to transform immunosuppressive strategies by enabling individualized treatment decisions in the pretransplant setting.

This study is not without limitations. First, immunosuppressive regimens were not standardized across sites. In addition, donor-specific antibodies monitoring was performed according to local clinical practice rather than as part of a study protocol, limiting uniformity in data collection. Similarly, indication biopsies were performed based on center-specific criteria, which may have introduced variability in diagnostic thresholds of clinical suspicion. Moreover, the relatively high observed AR rate (40%) likely reflects enrichment for higher-risk patients, the requirement for surveillance biopsies, and the inclusion of borderline rejection events in both pretransplant and post-transplant analyses, as well as inclusion of up to a 2-year time window for AR occurrence. Finally, this study focused on the 60 days post-transplant period as the most proximate and critically relevant window that recipient immune activation is most likely to manifest as EAR. Our aim was to assess whether pretransplant immune signatures could identify patients at heightened risk during this critical early phase. During this critical time, for-cause biopsies are performed to assess the condition of the allograft usually in response to clinical indicators and suspicion. Investigators were blinded to test results; however, the small number of for-cause biopsies and EAR events by 60 days should be considered when interpreting these findings. Collectively, although these factors may introduce heterogeneity, they also enhance the real-world relevance and generalizability of our findings.

In summary, we have identified and validated a 29-gene pretransplant RNA signature that accurately predicts the risk of EAR within 60 days post-transplant in patients receiving a DD kidney. This signature supports the use of PTRA as a precision medicine tool for tailoring immunosuppression to individual patients, with the goal of improving long-term graft health and reducing unnecessary treatment-related complications. Unlike other assessments, PTRA operates independently of donor characteristics, reinforcing the central role of the recipient's immune state in guiding transplant care decisions. Our findings support continued investigation of the PTRA score as a clinically actionable biomarker for pretransplant risk stratification and personalized immunosuppression.

## Supplementary Material

## Data Availability

Original data generated for the study will be made available upon reasonable request to the corresponding author. Data Type: Health Care Data; Clinical Trial Data. Reason for Restricted Access: Data are available on reasonable request because of proprietary and IP-related information.

## Declarative Statements

This study includes clinical experimentation and received Institutional Review Board or Ethics Committee approval. All patients provided written informed consent. This study includes clinical experimentation and complies with the Declaration of Helsinki. The clinical and research activities being reported are consistent with the Principles of the Declaration of Istanbul as outlined in the Declaration of Istanbul on Organ Trafficking and Transplant Tourism.

## Supplemental Material

Signature Gene Selection and Function Methodology.

Supplemental Figure 1. Identification of differentially expressed genes in pretransplant blood.

Supplemental Table 1. List of 29 genes included in PRTA signature assay.

Supplemental Figure 2. ROC for class 1 and class 2 PRA.

## References

[1] Meier-Kriesche HU Schold JD Srinivas TR Kaplan B. Lack of improvement in renal allograft survival despite a marked decrease in acute rejection rates over the most recent era. Am J Transplant. 2004;4(3):378–383. doi:10.1111/j.1600-6143.2004.00332.x 14961990

[2] Meier-Kriesche HU Schold JD Kaplan B. Long-term renal allograft survival: have we made significant progress or is it time to rethink our analytic and therapeutic strategies? Am J Transplant. 2004;4(8):1289–1295. doi:10.1111/j.1600-6143.2004.00515.x 15268730

[3] Poggio ED Augustine JJ Arrigain S Brennan DC Schold JD. Long-term kidney transplant graft survival—Making progress when most needed. Am J Transplant. 2021;21(8):2824–2832. doi:10.1111/ajt.16463 33346917

[4] Stegall MD Gaston RS Cosio FG Matas A. Through a glass darkly: seeking clarity in preventing late kidney transplant failure. J Am Soc Nephrol. 2015;26(1):20–29. doi:10.1681/ASN.2014040378 25097209 PMC4279746

[5] Meier-Kriesche HU Ojo AO Hanson JA, . Increased impact of acute rejection on chronic allograft failure in recent era. Transplantation. 2000;70(7):1098–1100. doi:10.1097/00007890-200010150-00018 11045649

[6] Dunn TB Noreen H Gillingham K, . Revisiting traditional risk factors for rejection and graft loss after kidney transplantation. Am J Transplant. 2011;11(10):2132–2143. doi:10.1111/J.1600-6143.2011.03640.X 21812918 PMC3184338

[7] Cole EH Johnston O Rose CL Gill JS. Impact of acute rejection and new-onset diabetes on long-term transplant graft and patient survival. Clin J Am Soc Nephrol. 2008;3(3):814–821. doi:10.2215/CJN.04681107 18322046 PMC2386715

[8] Gaston RS Fieberg A Hunsicker L, . Late graft failure after kidney transplantation as the consequence of late versus early events. Am J Transplant. 2018;18(5):1158–1167. doi:10.1111/ajt.14590 29139625

[9] Von Samson-Himmelstjerna FA Kakavand N Gleske C, . Potential and uncertainties of RejectClass in acute kidney graft dysfunction: an independent validation study. Transplantation. 2024;108(5):1228–1238. doi:10.1097/TP.0000000000004906 38196094 PMC11042522

[10] Seifert ME Agarwal G Bernard M, . Impact of subclinical borderline inflammation on kidney transplant outcomes. Transplant Direct. 2021;7(2):e663. doi:10.1097/TXD.0000000000001119 33511268 PMC7837932

[11] Cippà PE Schiesser M Ekberg H, . Risk stratification for rejection and infection after kidney transplantation. Clin J Am Soc Nephrol. 2015;10(12):2213–2220. doi:10.2215/CJN.01790215 26430088 PMC4670759

[12] Cosio FG Grande JP Wadei H Larson TS Griffin MD Stegall MD. Predicting subsequent decline in kidney allograft function from early surveillance biopsies. Am J Transplant. 2005;5(10):2464–2472. doi:10.1111/j.1600-6143.2005.01050.x 16162196

[13] Yabu JM Siebert JC Maecker HT. Immune profiles to predict response to desensitization therapy in highly HLA-sensitized kidney transplant candidates. PLoS One. 2016;11(4):e0153355. doi:10.1371/JOURNAL.PONE.0153355 27078882 PMC4831845

[14] Robertson H Kim HJ Li J, . Decoding the hallmarks of allograft dysfunction with a comprehensive pan-organ transcriptomic atlas. Nat Med. 2024;30(12):3748–3757. doi:10.1038/S41591-024-03030-6 38890530 PMC11645273

[15] Poggio ED Clemente M Hricik DE Heeger PS. Panel of reactive T cells as a measurement of primed cellular alloimmunity in kidney transplant candidates. J Am Soc Nephrol. 2006;17(2):564–572. doi:10.1681/ASN.2005030293 16382020

[16] Lebranchu Y Baan C Biancone L, . Pretransplant identification of acute rejection risk following kidney transplantation. Transpl Int. 2014;27(2):129–138. doi:10.1111/tri.12205 24118550

[17] Hricik DE Formica RN Nickerson P, . Adverse outcomes of tacrolimus withdrawal in immune-quiescent kidney transplant recipients. J Am Soc Nephrol. 2015;26(12):3114–3122. doi:10.1681/ASN.2014121234 25925687 PMC4657844

[18] Archer KJ Bardhi E Maluf DG, . Pretransplant kidney transcriptome captures intrinsic donor organ quality and predicts 24-month outcomes. Am J Transplant. 2022;22(11):2515–2528. doi:10.1111/ajt.17127 35730259 PMC9710201

[19] Puthumana J Hall IE Reese PP, . YKL-40 associates with renal recovery in deceased donor kidney transplantation. J Am Soc Nephrol. 2017;28(2):661–670. doi:10.1681/ASN.2016010091 27451287 PMC5280016

[20] Giangreco NP Lebreton G Restaino S, . Alterations in the kallikrein-kinin system predict death after heart transplant. Sci Rep. 2022;12(1):14167. doi:10.1038/S41598-022-18573-2 35986069 PMC9391369

[21] Augustine JJ Siu DS Clemente MJ Schulak JA Heeger PS Hricik DE. Pre‐transplant IFN‐γ ELISPOTs are associated with post‐transplant renal function in African American renal transplant recipients. Am J Transplant. 2005;5(8):1971–1975. doi:10.1111/J.1600-6143.2005.00958.X 15996247

[22] Bestard O Meneghini M Crespo E, . Preformed T cell alloimmunity and HLA eplet mismatch to guide immunosuppression minimization with tacrolimus monotherapy in kidney transplantation: results of the CELLIMIN trial. Am J Transplant. 2021;21(8):2833–2845. doi:10.1111/ajt.16563 33725408

[23] Zhang W Yi Z Wei C, . Pretransplant transcriptomic signature in peripheral blood predicts early acute rejection. JCI Insight. 2019;4(11):e127543. doi:10.1172/JCI.INSIGHT.127543 31167967 PMC6629121

[24] Bestard O Augustine J Wee A, . Prospective observational study to validate a next-generation sequencing blood RNA signature to predict early kidney transplant rejection. Am J Transplant. 2024;24(3):436–447. doi:10.1016/j.ajt.2023.09.021 38152017

[25] Nemati E Einollahi B Pezeshki ML Porfarziani V Fattahi MR. Does kidney transplantation with deceased or living donor affect graft survival? Nephrourol Mon. 2014;6(4):e12182. doi:10.5812/NUMONTHLY.12182 25695017 PMC4317718

[26] Toapanta N Comas J Revuelta I, . Benefits of living over deceased donor kidney transplantation in elderly recipients. A propensity score matched analysis of a large European Registry Cohort. Transpl Int. 2024;37:13452. doi:10.3389/ti.2024.13452 39263600 PMC11387891

[27] Loupy A Haas M Roufosse C, . The Banff 2019 Kidney Meeting Report (I): updates on and clarification of criteria for T cell– and antibody‐mediated rejection. Am J Transplant. 2020;20(9):2318–2331. doi:10.1111/AJT.15898 32463180 PMC7496245

[28] Newman AM Steen CB Liu CL, . Determining cell type abundance and expression from bulk tissues with digital cytometry. Nat Biotechnol. 2019;37(7):773–782. doi:10.1038/S41587-019-0114-2 31061481 PMC6610714

[29] Maas K Chan S Parker J, . Cutting edge: molecular portrait of human autoimmune disease. J Immunol. 2002;169(1):5–9. doi:10.4049/JIMMUNOL.169.1.5 12077221

[30] Mesko B Poliska S Nagy L. Gene expression profiles in peripheral blood for the diagnosis of autoimmune diseases. Trends Mol Med. 2011;17(4):223–233. doi:10.1016/j.molmed.2010.12.004 21388884

[31] Reis-Filho JS Pusztai L. Gene expression profiling in breast cancer: classification, prognostication, and prediction. Lancet. 2011;378(9805):1812–1823. doi:10.1016/S0140-6736(11)61539-0 22098854

[32] Vondran FWR Timrott K Kollrich S, . Pretransplant immune state defined by serum markers and alloreactivity predicts acute rejection after living donor kidney transplantation. Clin Transplant. 2014;28(9):968–979. doi:10.1111/ctr.12399 24931031

[33] Dedeoglu B Meijers RWJ Klepper M, Loss of CD28 on peripheral T cells decreases the risk for early acute rejection after kidney transplantation. PLoS One. 2016;11(3):e0150826. doi:10.1371/JOURNAL.PONE.0150826 26950734 PMC4780739

[34] Pontrelli P Rascio F Castellano G Grandaliano G Gesualdo L Stallone G. The role of natural killer cells in the immune response in kidney transplantation. Front Immunol. 2020;11:1454. doi:10.3389/fimmu.2020.01454 32793200 PMC7390843

[35] Duygu B Olieslagers TI Groeneweg M Voorter CEM Wieten L. HLA class I molecules as immune checkpoints for NK cell alloreactivity and anti-viral immunity in kidney transplantation. Front Immunol. 2021;12:680480. doi:10.3389/fimmu.2021.680480 34295330 PMC8290519

[36] Sprangers B Nair V Launay-Vacher V Riella LV Jhaveri KD. Risk factors associated with post–kidney transplant malignancies: an article from the cancer-kidney international network. Clin Kidney J. 2018;11(3):315–329. doi:10.1093/CKJ/SFX122 29942495 PMC6007332

[37] Taber DJ Gordon EJ Myaskovsky L, . Therapeutic needs in solid organ transplant recipients: the American society of transplantation patient survey. Am J Transplant. 2025;25(12):2565–2577. doi:10.1016/j.ajt.2025.07.2474 40744428

