# Supplementary material for: A Pre-Kidney Transplant Blood-Based Next-Generation Sequencing Assay to Predict Early Acute Rejection
Source: Kidney360. 2026 May 14;7(8):1792–806. doi: 10.34067/KID.0000001208 (PMC13567880; doi:10.34067/KID.0000001208)
Supplement: Supplementary file 1 [file kidney360-7-1792-s001.pdf]

ASN Journal Disclosure Form

As per ASN journal policy, I have disclosed any financial relationships or commitments I have held in the past 36 months as included below. I have listed my Current Employer below to indicate there is a relationship requiring disclosure. If no relationship exists, my Current Employer is not listed.

F. Alberici reports the following:
Employer: University of Brescia; Consultancy: CSL; Novartis; Alexion; Research Funding: Sanofi; and Honoraria: CSL; Novartis.

I understand that the information above will be published within the journal article, if accepted, and that failure to comply and/or to accurately and completely report the potential financial conflicts of interest could lead to the following: 1) Prior to publication, article rejection, or 2) Post-publication, sanctions ranging from, but not limited to, issuing a correction, reporting the inaccurate information to the authors' institution, banning authors from submitting work to ASN journals for varying lengths of time, and/or retraction of the published work.

Name: Federico Alberici
Manuscript ID: K360-2025-000962R2
Manuscript Title: Multi-center international study to validate a pre-transplant blood-based next-generation sequencing assay to predict early acute rejection post-kidney transplant
Date of Completion: February 9, 2026
Disclosure Updated Date: February 9, 2026

ASN American Society of Nephrology · JASN Journal of the American Society of Nephrology · KIDNEY360 Accessing Our World From Every Angle · CJASN Clinical Journal of the American Society of Nephrology

ASN Journal Disclosure Form

As per ASN journal policy, I have disclosed any financial relationships or commitments I have held in the past 36 months as included below. I have listed my Current Employer below to indicate there is a relationship requiring disclosure. If no relationship exists, my Current Employer is not listed.

M. Ansari reports the following:
Employer: Northwestern Memorial Hospital; Consultancy: AlloVir; Vertex; Verici Dx; Research Funding: Eurofins/Transplant Genomics; Verici Dx; AlloVir; Honoraria: AlloVir; Apellis; Vertex; Verici Dx; Patents or Royalties: Tract Therapeutics; Advisory or Leadership Role: AlloVir; Vertex; and Speakers Bureau: Apellis; Verici Dx.

I understand that the information above will be published within the journal article, if accepted, and that failure to comply and/or to accurately and completely report the potential financial conflicts of interest could lead to the following: 1) Prior to publication, article rejection, or 2) Post-publication, sanctions ranging from, but not limited to, issuing a correction, reporting the inaccurate information to the authors' institution, banning authors from submitting work to ASN journals for varying lengths of time, and/or retraction of the published work.

Name: Mohammed Javeed Ansari
Manuscript ID: K360-2025-000962R3
Manuscript Title: Multi-center international study to validate a pre-transplant blood-based next-generation sequencing assay to predict early acute rejection post-kidney transplant
Date of Completion: March 23, 2026
Disclosure Updated Date: March 23, 2026

ASN American Society of Nephrology | JASN Journal of the American Society of Nephrology | KIDNEY360 Accessing Our World From Every Angle | CJASN Clinical Journal of the American Society of Nephrology

ASN Journal Disclosure Form

As per ASN journal policy, I have disclosed any financial relationships or commitments I have held in the past 36 months as included below. I have listed my Current Employer below to indicate there is a relationship requiring disclosure. If no relationship exists, my Current Employer is not listed.

J. Augustine reports the following:
Employer: Cleveland Clinic Foundation

I understand that the information above will be published within the journal article, if accepted, and that failure to comply and/or to accurately and completely report the potential financial conflicts of interest could lead to the following: 1) Prior to publication, article rejection, or 2) Post-publication, sanctions ranging from, but not limited to, issuing a correction, reporting the inaccurate information to the authors' institution, banning authors from submitting work to ASN journals for varying lengths of time, and/or retraction of the published work.

Name: Joshua J. Augustine
Manuscript ID: K360-2025-000962R4
Manuscript Title: A pre-kidney transplant blood-based next-generation sequencing assay to predict early acute rejection
Date of Completion: April 15, 2026
Disclosure Updated Date: April 15, 2026

ASN Journal Disclosure Form

As per ASN journal policy, I have disclosed any financial relationships or commitments I have held in the past 36 months as included below. I have listed my Current Employer below to indicate there is a relationship requiring disclosure. If no relationship exists, my Current Employer is not listed.

O. Bestard reports the following:
Employer: Vall d'Hebron University Hospital; Research Funding: Takeda, Natera; and Advisory or Leadership Role: Hansa biopharma, Veloxis, Sanofi, Astellas, Eledon, Sobi.

I understand that the information above will be published within the journal article, if accepted, and that failure to comply and/or to accurately and completely report the potential financial conflicts of interest could lead to the following: 1) Prior to publication, article rejection, or 2) Post-publication, sanctions ranging from, but not limited to, issuing a correction, reporting the inaccurate information to the authors' institution, banning authors from submitting work to ASN journals for varying lengths of time, and/or retraction of the published work.

Name: Oriol Bestard
Manuscript ID: K360-2025-000962R3
Manuscript Title: Multi-center international study to validate a pre-transplant blood-based next-generation sequencing assay to predict early acute rejection post-kidney transplant
Date of Completion: March 26, 2026
Disclosure Updated Date: March 2, 2026

ASN Journal Disclosure Form

As per ASN journal policy, I have disclosed any financial relationships or commitments I have held in the past 36 months as included below. I have listed my Current Employer below to indicate there is a relationship requiring disclosure. If no relationship exists, my Current Employer is not listed.

C. Bhati reports the following:
Employer: University of Maryland; Consultancy: Intuitive Surgical; and Honoraria: Intuitive Surgical.

I understand that the information above will be published within the journal article, if accepted, and that failure to comply and/or to accurately and completely report the potential financial conflicts of interest could lead to the following: 1) Prior to publication, article rejection, or 2) Post-publication, sanctions ranging from, but not limited to, issuing a correction, reporting the inaccurate information to the authors' institution, banning authors from submitting work to ASN journals for varying lengths of time, and/or retraction of the published work.

Name: Chandra Bhati
Manuscript ID: K360-2025-000962R1
Manuscript Title: Multi-center international study to validate a pre-transplant blood-based next-generation sequencing assay to predict early acute rejection post-kidney transplant
Date of Completion: December 2, 2025
Disclosure Updated Date: December 2, 2025

ASN American Society of Nephrology | JASN Journal of the American Society of Nephrology | KIDNEY360 Accessing Our World From Every Angle | CJASN Clinical Journal of the American Society of Nephrology

ASN Journal Disclosure Form

As per ASN journal policy, I have disclosed any financial relationships or commitments I have held in the past 36 months as included below. I have listed my Current Employer below to indicate there is a relationship requiring disclosure. If no relationship exists, my Current Employer is not listed.

A. Chang reports the following:
Employer: University of Chicago Medicine; Consultancy: Alexion; Novartis, Apellis, Otsuka, Amgen, Genentech, Calliditas, iMDx, VericiDx; Honoraria: Alexion Pharmaceuticals, Otsuka, Novartis, Apellis; Advisory or Leadership Role: Editorial Board - Archives of Pathology and Laboratory Medicine (unpaid); Chicago Pathology Society - Board of Directors (unpaid); Speakers Bureau: Alexion Pharmaceuticals, Otsuka, Novartis, Apellis; and Other Interests or Relationships: Elsevier - textbook royalties.

I understand that the information above will be published within the journal article, if accepted, and that failure to comply and/or to accurately and completely report the potential financial conflicts of interest could lead to the following: 1) Prior to publication, article rejection, or 2) Post-publication, sanctions ranging from, but not limited to, issuing a correction, reporting the inaccurate information to the authors' institution, banning authors from submitting work to ASN journals for varying lengths of time, and/or retraction of the published work.

Name: Anthony Chang
Manuscript ID: K360-2025-000962R4
Manuscript Title: A pre-kidney transplant blood-based next-generation sequencing assay to predict early acute rejection
Date of Completion: April 14, 2026
Disclosure Updated Date: April 14, 2026

ASN American Society of Nephrology | JASN Journal of the American Society of Nephrology | KIDNEY360 Accessing Our World From Every Angle | CJASN Clinical Journal of the American Society of Nephrology

ASN Journal Disclosure Form

As per ASN journal policy, I have disclosed any financial relationships or commitments I have held in the past 36 months as included below. I have listed my Current Employer below to indicate there is a relationship requiring disclosure. If no relationship exists, my Current Employer is not listed.

A. Cherukuri reports the following:
Employer: University of Pittsburgh Medical Center; Patents or Royalties: Pending patent for the use of b cell cytokines as biomarker for rejection; and Advisory or Leadership Role: Aravind Cherukuri, Verici diagnostics 2026.

I understand that the information above will be published within the journal article, if accepted, and that failure to comply and/or to accurately and completely report the potential financial conflicts of interest could lead to the following: 1) Prior to publication, article rejection, or 2) Post-publication, sanctions ranging from, but not limited to, issuing a correction, reporting the inaccurate information to the authors' institution, banning authors from submitting work to ASN journals for varying lengths of time, and/or retraction of the published work.

Name: Aravind Cherukuri
Manuscript ID: K360-2025-000962R2
Manuscript Title: Multi-center international study to validate a pre-transplant blood-based next-generation sequencing assay to predict early acute rejection post-kidney transplant
Date of Completion: April 3, 2026
Disclosure Updated Date: April 3, 2026

ASN Journal Disclosure Form

As per ASN journal policy, I have disclosed any financial relationships or commitments I have held in the past 36 months as included below. I have listed my Current Employer below to indicate there is a relationship requiring disclosure. If no relationship exists, my Current Employer is not listed.

G. Comai has nothing to disclose.

I understand that the information above will be published within the journal article, if accepted, and that failure to comply and/or to accurately and completely report the potential financial conflicts of interest could lead to the following: 1) Prior to publication, article rejection, or 2) Post-publication, sanctions ranging from, but not limited to, issuing a correction, reporting the inaccurate information to the authors' institution, banning authors from submitting work to ASN journals for varying lengths of time, and/or retraction of the published work.

Name: Giorgia Comai
Manuscript ID: K360-2025-000962R2
Manuscript Title: Multi-center international study to validate a pre-transplant blood-based next-generation sequencing assay to predict early acute rejection post-kidney transplant
Date of Completion: March 20, 2026
Disclosure Updated Date: March 20, 2026

ASN American Society of Nephrology JASN Journal of the American Society of Nephrology KIDNEY360 Accessing Our World From Every Angle CJASN Clinical Journal of the American Society of Nephrology

ASN Journal Disclosure Form

As per ASN journal policy, I have disclosed any financial relationships or commitments I have held in the past 36 months as included below. I have listed my Current Employer below to indicate there is a relationship requiring disclosure. If no relationship exists, my Current Employer is not listed.

B. Concepcion reports the following:
Employer: University of Chicago;; Consultancy: Ipsen pharmaceuticals; Honoraria: Veloxis, Novartis; Patents or Royalties: Springer; and Advisory or Leadership Role: Editorial board member of AJKD; NKF health equity advisory committee.

I understand that the information above will be published within the journal article, if accepted, and that failure to comply and/or to accurately and completely report the potential financial conflicts of interest could lead to the following: 1) Prior to publication, article rejection, or 2) Post-publication, sanctions ranging from, but not limited to, issuing a correction, reporting the inaccurate information to the authors' institution, banning authors from submitting work to ASN journals for varying lengths of time, and/or retraction of the published work.

Name: Beatrice P. Concepcion
Manuscript ID: K360-2025-000962R1
Manuscript Title: Multi-center international study to validate a pre-transplant blood-based next-generation sequencing assay to predict early acute rejection post-kidney transplant
Date of Completion: December 3, 2025
Disclosure Updated Date: October 15, 2025

ASN
American Society of Nephrology
JASN
Journal of the American Society of Nephrology
KIDNEY360
Accessing Our World From Every Angle
CJASN
Clinical Journal of the American Society of Nephrology

ASN Journal Disclosure Form

As per ASN journal policy, I have disclosed any financial relationships or commitments I have held in the past 36 months as included below. I have listed my Current Employer below to indicate there is a relationship requiring disclosure. If no relationship exists, my Current Employer is not listed.

P. Connolly reports the following:
Employer: Verici Dx Inc, Spouse - Merryman Farr Mechanical; Consultancy: Amplified Sciences; Ownership Interest: Verici Dx Inc, Renalytix, Outset Medical; Patents or Royalties: Verici Dx; and Advisory or Leadership Role: Verici Dx Inc (employee), Amplified Sciences ($5,000 annual for SAB).

I understand that the information above will be published within the journal article, if accepted, and that failure to comply and/or to accurately and completely report the potential financial conflicts of interest could lead to the following: 1) Prior to publication, article rejection, or 2) Post-publication, sanctions ranging from, but not limited to, issuing a correction, reporting the inaccurate information to the authors' institution, banning authors from submitting work to ASN journals for varying lengths of time, and/or retraction of the published work.

Name: Patricia Connolly
Manuscript ID: K360-2025-000962R3
Manuscript Title: Multi-center international study to validate a pre-transplant blood-based next-generation sequencing assay to predict early acute rejection post-kidney transplant
Date of Completion: March 23, 2026
Disclosure Updated Date: March 23, 2026

ASN American Society of Nephrology | JASN Journal of the American Society of Nephrology | KIDNEY360 Accessing Our World From Every Angle | CJASN Clinical Journal of the American Society of Nephrology

ASN Journal Disclosure Form

As per ASN journal policy, I have disclosed any financial relationships or commitments I have held in the past 36 months as included below. I have listed my Current Employer below to indicate there is a relationship requiring disclosure. If no relationship exists, my Current Employer is not listed.

P. Di Cocco reports the following:
Employer: University of Illinois at Chicago

I understand that the information above will be published within the journal article, if accepted, and that failure to comply and/or to accurately and completely report the potential financial conflicts of interest could lead to the following: 1) Prior to publication, article rejection, or 2) Post-publication, sanctions ranging from, but not limited to, issuing a correction, reporting the inaccurate information to the authors' institution, banning authors from submitting work to ASN journals for varying lengths of time, and/or retraction of the published work.

Name: Pierpaolo Di Cocco
Manuscript ID: K360-2025-000962R2
Manuscript Title: "Multi-center international study to validate a pre-transplant blood-based next-generation sequencing assay to predict early acute rejection post-kidney transplant
Date of Completion: March 25, 2026
Disclosure Updated Date: March 25, 2026

ASN
American Society of Nephrology
JASN
Journal of the American Society of Nephrology
KIDNEY360
Accessing Our World From Every Angle
CJASN
Clinical Journal of the American Society of Nephrology

ASN Journal Disclosure Form

As per ASN journal policy, I have disclosed any financial relationships or commitments I have held in the past 36 months as included below. I have listed my Current Employer below to indicate there is a relationship requiring disclosure. If no relationship exists, my Current Employer is not listed.

M. Donovan reports the following:
Employer: Renalytix;; Consultancy: VericiDx; PreciseDx; Ownership Interest: Renalytix; VericiDx; PreciseDx; Patents or Royalties: PreciseDx; and Advisory or Leadership Role: Renalytix; VericiDx (60,000.00 / year).

I understand that the information above will be published within the journal article, if accepted, and that failure to comply and/or to accurately and completely report the potential financial conflicts of interest could lead to the following: 1) Prior to publication, article rejection, or 2) Post-publication, sanctions ranging from, but not limited to, issuing a correction, reporting the inaccurate information to the authors' institution, banning authors from submitting work to ASN journals for varying lengths of time, and/or retraction of the published work.

Name: Michael J. Donovan
Manuscript ID: K360-2025-000962R2
Manuscript Title: "Multi-center international study to validate a pre-transplant blood-based next-generation sequencing assay to predict early acute rejection post-kidney transplant,"
Date of Completion: February 3, 2026
Disclosure Updated Date: February 3, 2026

ASN
American Society of Nephrology
JASN
Journal of the American Society of Nephrology
KIDNEY360
Accessing Our World From Every Angle
CJASN
Clinical Journal of the American Society of Nephrology

ASN Journal Disclosure Form

As per ASN journal policy, I have disclosed any financial relationships or commitments I have held in the past 36 months as included below. I have listed my Current Employer below to indicate there is a relationship requiring disclosure. If no relationship exists, my Current Employer is not listed.

N. Doshi reports the following:
Employer: Verici Dx;; Ownership Interest: Verici Dx; and Patents or Royalties: Verici Dx.

I understand that the information above will be published within the journal article, if accepted, and that failure to comply and/or to accurately and completely report the potential financial conflicts of interest could lead to the following: 1) Prior to publication, article rejection, or 2) Post-publication, sanctions ranging from, but not limited to, issuing a correction, reporting the inaccurate information to the authors' institution, banning authors from submitting work to ASN journals for varying lengths of time, and/or retraction of the published work.

Name: Nehal Doshi
Manuscript ID: K360-2025-000962R2
Manuscript Title: Multi-center international study to validate a pre-transplant blood-based next-generation sequencing assay to predict early acute rejection post-kidney transplant
Date of Completion: February 11, 2026
Disclosure Updated Date: February 11, 2026

ASN American Society of Nephrology | JASN Journal of the American Society of Nephrology | KIDNEY360 Accessing Our World From Every Angle | CJASN Clinical Journal of the American Society of Nephrology

ASN Journal Disclosure Form

As per ASN journal policy, I have disclosed any financial relationships or commitments I have held in the past 36 months as included below. I have listed my Current Employer below to indicate there is a relationship requiring disclosure. If no relationship exists, my Current Employer is not listed.

L. Gallon reports the following:
Employer: University of Illinois; Consultancy: Verici; Honoraria: Calliditas; Natera; Thermo-Fisher;; and Advisory or Leadership Role: Verici.

I understand that the information above will be published within the journal article, if accepted, and that failure to comply and/or to accurately and completely report the potential financial conflicts of interest could lead to the following: 1) Prior to publication, article rejection, or 2) Post-publication, sanctions ranging from, but not limited to, issuing a correction, reporting the inaccurate information to the authors' institution, banning authors from submitting work to ASN journals for varying lengths of time, and/or retraction of the published work.

Name: Lorenzo G. Gallon
Manuscript ID: K360-2025-000962R4
Manuscript Title: A pre-kidney transplant blood-based next-generation sequencing assay to predict early acute rejection
Date of Completion: April 14, 2026
Disclosure Updated Date: November 29, 2025

ASN Journal Disclosure Form

As per ASN journal policy, I have disclosed any financial relationships or commitments I have held in the past 36 months as included below. I have listed my Current Employer below to indicate there is a relationship requiring disclosure. If no relationship exists, my Current Employer is not listed.

E. Gessaroli reports the following:
Employer: Department of Medicine, University Illinois at Chicago; Department of Medical and Surgical Sciences (DIMEC), Alma Mater Studiorum - University of Bologna; and Consultancy: Sanofi (ended 12/2025) Manuscript consultancy.

I understand that the information above will be published within the journal article, if accepted, and that failure to comply and/or to accurately and completely report the potential financial conflicts of interest could lead to the following: 1) Prior to publication, article rejection, or 2) Post-publication, sanctions ranging from, but not limited to, issuing a correction, reporting the inaccurate information to the authors' institution, banning authors from submitting work to ASN journals for varying lengths of time, and/or retraction of the published work.

Name: Elisa Gessaroli
Manuscript ID: K360-2025-000962R2
Manuscript Title: Multi-center international study to validate a pre-transplant blood-based next-generation sequencing assay to predict early acute rejection post-kidney transplant
Date of Completion: February 6, 2026
Disclosure Updated Date: February 6, 2026

ASN
American Society of Nephrology
JASN
Journal of the American Society of Nephrology
KIDNEY360
Accessing Our World From Every Angle
CJASN
Clinical Journal of the American Society of Nephrology

ASN Journal Disclosure Form

As per ASN journal policy, I have disclosed any financial relationships or commitments I have held in the past 36 months as included below. I have listed my Current Employer below to indicate there is a relationship requiring disclosure. If no relationship exists, my Current Employer is not listed.

There is no minimum financial threshold; individuals must disclose all financial relationships, regardless of the amount, with ineligible companies. Individuals must disclose for every category below, regardless of their view of the relevance of the relationship to the activity. ("*Ineligible companies" are those whose primary business is producing, marketing, selling, re-selling, or distributing health care products used by or on patients.*)

Date: May 8, 2026

Author Name: Michael W. Kattan

Manuscript ID: K360-2025-000962R3

Manuscript Title: Multi-center international study to validate a pre-transplant blood-based next-generation sequencing assay to predict early acute rejection post-kidney transplant

Disclosure Statement: *(including all categories below*)*

Dr. Kattan was a paid consultant to VericiDx during the development of the MS.

***Categories:** Employer; Ownership Interest; Consultancy; Research Funding; Honoraria; Patents or Royalties; Advisory or Leadership Role; Speakers Bureau; and Other Interests or Relationships

Author Acknowledgment: I understand that the information above will be published within the journal article, if accepted, and that failure to comply and/or to accurately and completely report the potential financial conflicts of interest could lead to the following: 1) Prior to publication, article rejection, or 2) Post-publication, sanctions ranging from, but not limited to, issuing a correction, reporting the inaccurate information to the authors' institution, banning authors from submitting work to ASN journals for varying lengths of time, and/or retraction of the published work.

Author Name and/or Initials: Michael W. Kattan

ASN
American Society of Nephrology
JASN
Journal of the American Society of Nephrology
KIDNEY360
Accessing Our World From Every Angle
CJASN
Clinical Journal of the American Society of Nephrology

ASN Journal Disclosure Form

As per ASN journal policy, I have disclosed any financial relationships or commitments I have held in the past 36 months as included below. I have listed my Current Employer below to indicate there is a relationship requiring disclosure. If no relationship exists, my Current Employer is not listed.

N. Leca reports the following:
Employer: University of Washington; Consultancy: Alexion, Natera, Novartis, Travere, Sanofi, Veloxis; and Research Funding: Novartis CareDx Natera Transplant Genomics Verici MEMO Hansa Alexion Biogen ArgenX.

I understand that the information above will be published within the journal article, if accepted, and that failure to comply and/or to accurately and completely report the potential financial conflicts of interest could lead to the following: 1) Prior to publication, article rejection, or 2) Post-publication, sanctions ranging from, but not limited to, issuing a correction, reporting the inaccurate information to the authors' institution, banning authors from submitting work to ASN journals for varying lengths of time, and/or retraction of the published work.

Name: Nicolae Leca
Manuscript ID: K360-2025-000962R2
Manuscript Title: Multi-center international study to validate a pre-transplant blood-based next-generation sequencing assay to predict early acute rejection post-kidney transplant
Date of Completion: March 20, 2026
Disclosure Updated Date: December 2, 2025

ASN American Society of Nephrology · JASN Journal of the American Society of Nephrology · KIDNEY360 Accessing Our World From Every Angle · CJASN Clinical Journal of the American Society of Nephrology

ASN Journal Disclosure Form

As per ASN journal policy, I have disclosed any financial relationships or commitments I have held in the past 36 months as included below. I have listed my Current Employer below to indicate there is a relationship requiring disclosure. If no relationship exists, my Current Employer is not listed.

D. Maluf reports the following:
Employer: University of Maryland School of Medicine

I understand that the information above will be published within the journal article, if accepted, and that failure to comply and/or to accurately and completely report the potential financial conflicts of interest could lead to the following: 1) Prior to publication, article rejection, or 2) Post-publication, sanctions ranging from, but not limited to, issuing a correction, reporting the inaccurate information to the authors' institution, banning authors from submitting work to ASN journals for varying lengths of time, and/or retraction of the published work.

Name: Daniel G. Maluf
Manuscript ID: K360-2025-000962R2
Manuscript Title: Multi-center international study to validate a pre-transplant blood-based next-generation sequencing assay to predict early acute rejection post-kidney transplant
Date of Completion: March 20, 2026
Disclosure Updated Date: March 20, 2026

ASN American Society of Nephrology | JASN Journal of the American Society of Nephrology | KIDNEY360 Accessing Our World From Every Angle | CJASN Clinical Journal of the American Society of Nephrology

ASN Journal Disclosure Form

As per ASN journal policy, I have disclosed any financial relationships or commitments I have held in the past 36 months as included below. I have listed my Current Employer below to indicate there is a relationship requiring disclosure. If no relationship exists, my Current Employer is not listed.

R. Mannon reports the following:
Consultancy: Alexion (Relationship has not ended); Biogen (Relationship has not ended); CSL-Behring (Relationship ended on 12/31/2025); ECOR1 (Relationship ended on 8/6/2025); Eledon (Relationship has not ended); Hansa (Relationship has not ended); Sanofi (Relationship has not ended); Third Arc Bio (Relationship ended on 1/31/2025); Vericii Dx (Relationship has not ended); Memo Therapeutics (Relationship has not ended); and Advisory or Leadership Role: Hennepin Healthcare Research Institute (Relationship has not ended); AST/ASTS (Relationship has not ended).

I understand that the information above will be published within the journal article, if accepted, and that failure to comply and/or to accurately and completely report the potential financial conflicts of interest could lead to the following: 1) Prior to publication, article rejection, or 2) Post-publication, sanctions ranging from, but not limited to, issuing a correction, reporting the inaccurate information to the authors' institution, banning authors from submitting work to ASN journals for varying lengths of time, and/or retraction of the published work.

Name: Roslyn B. Mannon
Manuscript ID: for K360-2025-000962R2
Manuscript Title: Multi-center international study to validate a pre-transplant blood-based next-generation sequencing assay to predict early acute rejection post-kidney transplant
Date of Completion: March 20, 2026
Disclosure Updated Date: March 6, 2026

ASN
American Society of Nephrology
JASN
Journal of the American Society of Nephrology
KIDNEY360
Accessing Our World From Every Angle
CJASN
Clinical Journal of the American Society of Nephrology

ASN Journal Disclosure Form

As per ASN journal policy, I have disclosed any financial relationships or commitments I have held in the past 36 months as included below. I have listed my Current Employer below to indicate there is a relationship requiring disclosure. If no relationship exists, my Current Employer is not listed.

E. Poggio reports the following:
Employer: Cleveland Clinic; Consultancy: CareDx, Transplant Genomics, Natera, Horizon, ThermoFisher, Veloxis, Sanofi; and Honoraria: CareDx, Transplant Genomics, Natera, Horizon, ThermoFisher, Veloxis, Sanofi.

I understand that the information above will be published within the journal article, if accepted, and that failure to comply and/or to accurately and completely report the potential financial conflicts of interest could lead to the following: 1) Prior to publication, article rejection, or 2) Post-publication, sanctions ranging from, but not limited to, issuing a correction, reporting the inaccurate information to the authors' institution, banning authors from submitting work to ASN journals for varying lengths of time, and/or retraction of the published work.

Name: Emilio D. Poggio
Manuscript ID: K360-2025-000962R2
Manuscript Title: Multi-center international study to validate a pre-transplant blood-based next-generation sequencing assay to predict early acute rejection post-kidney transplant
Date of Completion: March 20, 2026
Disclosure Updated Date: February 19, 2026

ASN American Society of Nephrology | JASN Journal of the American Society of Nephrology | KIDNEY360 Accessing Our World From Every Angle | CJASN Clinical Journal of the American Society of Nephrology

ASN Journal Disclosure Form

As per ASN journal policy, I have disclosed any financial relationships or commitments I have held in the past 36 months as included below. I have listed my Current Employer below to indicate there is a relationship requiring disclosure. If no relationship exists, my Current Employer is not listed.

R. Prashar reports the following:
Employer: Henry Ford hospital

I understand that the information above will be published within the journal article, if accepted, and that failure to comply and/or to accurately and completely report the potential financial conflicts of interest could lead to the following: 1) Prior to publication, article rejection, or 2) Post-publication, sanctions ranging from, but not limited to, issuing a correction, reporting the inaccurate information to the authors' institution, banning authors from submitting work to ASN journals for varying lengths of time, and/or retraction of the published work.

Name: Rohini Prashar
Manuscript ID: K360-2025-000962R2
Manuscript Title: Multi-center international study to validate a pre-transplant blood-based next-generation sequencing assay to predict early acute rejection post-kidney transplant,
Date of Completion: March 20, 2026
Disclosure Updated Date: November 24, 2025

ASN American Society of Nephrology · JASN Journal of the American Society of Nephrology · KIDNEY360 Accessing Our World From Every Angle · CJASN Clinical Journal of the American Society of Nephrology

ASN Journal Disclosure Form

As per ASN journal policy, I have disclosed any financial relationships or commitments I have held in the past 36 months as included below. I have listed my Current Employer below to indicate there is a relationship requiring disclosure. If no relationship exists, my Current Employer is not listed.

A. Rose reports the following:
Employer: Verici Dx; and Ownership Interest: Verici Dx.

I understand that the information above will be published within the journal article, if accepted, and that failure to comply and/or to accurately and completely report the potential financial conflicts of interest could lead to the following: 1) Prior to publication, article rejection, or 2) Post-publication, sanctions ranging from, but not limited to, issuing a correction, reporting the inaccurate information to the authors' institution, banning authors from submitting work to ASN journals for varying lengths of time, and/or retraction of the published work.

Name: Angela Rose
Manuscript ID: K360-2025-000962R1
Manuscript Title: Multi-center international study to validate a pre-transplant blood-based next-generation sequencing assay to predict early acute rejection post-kidney transplant,
Date of Completion: January 12, 2026
Disclosure Updated Date: January 12, 2026

ASN
American Society of Nephrology
JASN
Journal of the American Society of Nephrology
KIDNEY360
Accessing Our World From Every Angle
CJASN
Clinical Journal of the American Society of Nephrology

ASN Journal Disclosure Form

As per ASN journal policy, I have disclosed any financial relationships or commitments I have held in the past 36 months as included below. I have listed my Current Employer below to indicate there is a relationship requiring disclosure. If no relationship exists, my Current Employer is not listed.

L. Rostaing reports the following:
Employer: Pôle DIGIDUNE; Consultancy: BMS;; Honoraria: BMS/ASTELLAS/MSD/Astra-Zeneca/Alexion; and Advisory or Leadership Role: BMS / MSD.

I understand that the information above will be published within the journal article, if accepted, and that failure to comply and/or to accurately and completely report the potential financial conflicts of interest could lead to the following: 1) Prior to publication, article rejection, or 2) Post-publication, sanctions ranging from, but not limited to, issuing a correction, reporting the inaccurate information to the authors' institution, banning authors from submitting work to ASN journals for varying lengths of time, and/or retraction of the published work.

Name: Lionel Rostaing
Manuscript ID: K360-2025-000962R4
Manuscript Title: A pre-kidney transplant blood-based next-generation sequencing assay to predict early acute rejection
Date of Completion: April 14, 2026
Disclosure Updated Date: April 14, 2026

ASN American Society of Nephrology | JASN Journal of the American Society of Nephrology | KIDNEY360 Accessing Our World From Every Angle | CJASN Clinical Journal of the American Society of Nephrology

ASN Journal Disclosure Form

As per ASN journal policy, I have disclosed any financial relationships or commitments I have held in the past 36 months as included below. I have listed my Current Employer below to indicate there is a relationship requiring disclosure. If no relationship exists, my Current Employer is not listed.

D. Rothstein reports the following:
Employer: University of Pittsburgh; Consultancy: CSL Behring; Ownership Interest: Signal LTD; and Research Funding: Verici.

I understand that the information above will be published within the journal article, if accepted, and that failure to comply and/or to accurately and completely report the potential financial conflicts of interest could lead to the following: 1) Prior to publication, article rejection, or 2) Post-publication, sanctions ranging from, but not limited to, issuing a correction, reporting the inaccurate information to the authors' institution, banning authors from submitting work to ASN journals for varying lengths of time, and/or retraction of the published work.

Name: David M. Rothstein
Manuscript ID: K360-2025-000962R3
Manuscript Title: Multi-center international study to validate a pre-transplant blood-based next-generation sequencing assay to predict early acute rejection post-kidney transplant
Date of Completion: March 24, 2026
Disclosure Updated Date: March 24, 2026

ASN American Society of Nephrology | JASN Journal of the American Society of Nephrology | KIDNEY360 Accessing Our World From Every Angle | CJASN Clinical Journal of the American Society of Nephrology

ASN Journal Disclosure Form

As per ASN journal policy, I have disclosed any financial relationships or commitments I have held in the past 36 months as included below. I have listed my Current Employer below to indicate there is a relationship requiring disclosure. If no relationship exists, my Current Employer is not listed.

F. Salem reports the following:
Employer: Mayo Clinic, FL

I understand that the information above will be published within the journal article, if accepted, and that failure to comply and/or to accurately and completely report the potential financial conflicts of interest could lead to the following: 1) Prior to publication, article rejection, or 2) Post-publication, sanctions ranging from, but not limited to, issuing a correction, reporting the inaccurate information to the authors' institution, banning authors from submitting work to ASN journals for varying lengths of time, and/or retraction of the published work.

Name: Fadi E. Salem
Manuscript ID: K360-2025-000962R2
Manuscript Title: Multi-center international study to validate a pre-transplant blood-based next-generation sequencing assay to predict early acute rejection post-kidney transplant
Date of Completion: April 3, 2026
Disclosure Updated Date: April 3, 2026

ASN
American Society of Nephrology
JASN
Journal of the American Society of Nephrology
KIDNEY360
Accessing Our World From Every Angle
CJASN
Clinical Journal of the American Society of Nephrology

ASN Journal Disclosure Form

As per ASN journal policy, I have disclosed any financial relationships or commitments I have held in the past 36 months as included below. I have listed my Current Employer below to indicate there is a relationship requiring disclosure. If no relationship exists, my Current Employer is not listed.

M. Samaniego-Picota has nothing to disclose.

I understand that the information above will be published within the journal article, if accepted, and that failure to comply and/or to accurately and completely report the potential financial conflicts of interest could lead to the following: 1) Prior to publication, article rejection, or 2) Post-publication, sanctions ranging from, but not limited to, issuing a correction, reporting the inaccurate information to the authors' institution, banning authors from submitting work to ASN journals for varying lengths of time, and/or retraction of the published work.

Name: Milagros D. Samaniego-Picota
Manuscript ID: K360-2025-000962R3
Manuscript Title: e ("Multi-center international study to validate a pre-transplant blood-based next-generation sequencing assay to predict early acute rejection post-kidney transplant"
Date of Completion: March 31, 2026
Disclosure Updated Date: March 31, 2026

ASN Journal Disclosure Form

As per ASN journal policy, I have disclosed any financial relationships or commitments I have held in the past 36 months as included below. I have listed my Current Employer below to indicate there is a relationship requiring disclosure. If no relationship exists, my Current Employer is not listed.

J. Schold reports the following:
Employer: University of Colorado; Consultancy: Sanofi Corporation, Veloxis, Verici Dx and eGenesis; Research Funding: One Legacy Foundation, NIH/NIDDK, Kamada, Donor Alliance Foundation, Department of Defense, National Institutes of Health/NHLBI; Honoraria: Sanofi Inc; Advisory or Leadership Role: Data Safety Monitoring Board Member - Bristol Myers Squibb; ; Chair UNOS Data Advisory Committee; Scientific Registry of Transplant Recipients Review Committee, Secretary - American Society of Transplantation; and Speakers Bureau: Sanofi.

I understand that the information above will be published within the journal article, if accepted, and that failure to comply and/or to accurately and completely report the potential financial conflicts of interest could lead to the following: 1) Prior to publication, article rejection, or 2) Post-publication, sanctions ranging from, but not limited to, issuing a correction, reporting the inaccurate information to the authors' institution, banning authors from submitting work to ASN journals for varying lengths of time, and/or retraction of the published work.

Name: Jesse D. Schold
Manuscript ID: K360-2025-000962R1
Manuscript Title: Multi-center international study to validate a pre-transplant blood-based next-generation sequencing assay to predict early acute rejection post-kidney transplant
Date of Completion: December 2, 2025
Disclosure Updated Date: August 13, 2025

ASN American Society of Nephrology | JASN Journal of the American Society of Nephrology | KIDNEY360 Accessing Our World From Every Angle | CJASN Clinical Journal of the American Society of Nephrology

ASN Journal Disclosure Form

As per ASN journal policy, I have disclosed any financial relationships or commitments I have held in the past 36 months as included below. I have listed my Current Employer below to indicate there is a relationship requiring disclosure. If no relationship exists, my Current Employer is not listed.

S. Shawar reports the following:
Employer: Vanderbilt University Medical Center

I understand that the information above will be published within the journal article, if accepted, and that failure to comply and/or to accurately and completely report the potential financial conflicts of interest could lead to the following: 1) Prior to publication, article rejection, or 2) Post-publication, sanctions ranging from, but not limited to, issuing a correction, reporting the inaccurate information to the authors' institution, banning authors from submitting work to ASN journals for varying lengths of time, and/or retraction of the published work.

Name: Saed Shawar
Manuscript ID: K360-2025-000962R3
Manuscript Title: Multi-center international study to validate a pre-transplant blood-based next-generation sequencing assay to predict early acute rejection post-kidney transplant
Date of Completion: March 23, 2026
Disclosure Updated Date: March 23, 2026

ASN
American Society of Nephrology
JASN
Journal of the American Society of Nephrology
KIDNEY360
Accessing Our World From Every Angle
CJASN
Clinical Journal of the American Society of Nephrology

ASN Journal Disclosure Form

As per ASN journal policy, I have disclosed any financial relationships or commitments I have held in the past 36 months as included below. I have listed my Current Employer below to indicate there is a relationship requiring disclosure. If no relationship exists, my Current Employer is not listed.

A. Wee reports the following:
Employer: Cleveland Clinic; and Advisory or Leadership Role: Life Banc - Interim Medical Director - paid.

I understand that the information above will be published within the journal article, if accepted, and that failure to comply and/or to accurately and completely report the potential financial conflicts of interest could lead to the following: 1) Prior to publication, article rejection, or 2) Post-publication, sanctions ranging from, but not limited to, issuing a correction, reporting the inaccurate information to the authors' institution, banning authors from submitting work to ASN journals for varying lengths of time, and/or retraction of the published work.

Name: Alvin Wee
Manuscript ID: K360-2025-000962R2
Manuscript Title: Multi-center international study to validate a pre-transplant blood-based next-generation sequencing assay to predict early acute rejection post-kidney transplant
Date of Completion: March 26, 2026
Disclosure Updated Date: December 1, 2025
