# Supplementary material for: A Pre-Kidney Transplant Blood-Based Next-Generation Sequencing Assay to Predict Early Acute Rejection
Source: Kidney360. 2026 May 14;7(8):1792–806. doi: 10.34067/KID.0000001208 (PMC13567880; doi:10.34067/KID.0000001208)
Supplement: Supplementary file 2 [file kidney360-7-1792-s002.pdf]

Multi-center international study to validate a pre-transplant blood-based next-generation sequencing assay to predict early acute rejection post-kidney transplant

Beatrice P. Concepcion, Oriol Bestard, Milagros Samaniego-Picota, Rohini Prashar, M. Javeed Ansari, Giorgia Comai, Elisa Gessaroli, Lionel Rostaing, Federico Alberici, Roslyn B. Mannon, Joshua Augustine, Alvin Wee, Emilio Poggio, Pierpaolo DiCocco, Nicholae Leca, Saed Shawar, Chandra Bhati, Daniel Maluf, David M. Rothstein, Aravind Cherukuri, Anthony Chang, Fadi Salem, Michael W. Kattan, Jesse D. Schold, Patricia Connolly, Angela Rose, Nehal Doshi, Lorenzo Gallon, Michael J. Donovan

Supplemental Materials

1. Signature gene selection and function methodology
2. Supplemental Figure 1: Identification of differentially expressed genes in pre-transplant blood
3. Supplemental Table 1: List of 29 genes included in PRTA signature assay
4. Supplemental Figure 2: ROC curve for Class I and Class II PRA

1. Signature gene selection and function methodology

Good quality sequencing reads were trimmed and pre-filtered prior to downstream processing to ensure reliable gene-level quantification. For the discovery of candidate genes predictive of early acute rejection (EAR), genes falling within the target expression range in the discovery set were retained, while those outside the range were excluded. This filtering process yielded approximately 11,000 genes for further evaluation.

Differential expression analysis was performed using three established RNA-seq frameworks: DESeq2, edgeR, and limma-voom. Each was implemented with its recommended workflow, including normalization, dispersion, and variance modeling. From the filtered gene feature set, we applied recursive feature elimination (RFE) using shrinkage discriminant analysis (SDA) to identify the subset of genes most informative for early acute rejection (**Supplemental Figure 1**).

To further refine the feature set, we prioritized gene features with the highest predictive value for EAR area under the receiver operating characteristic (AUROC) curve in the training set under cross validation. Several classifier models were evaluated, including random forests, shrinkage discriminant analysis, and logistic regression. The final classifier was prespecified as a ridge-regularized (L2) logistic regression model with balanced class weights, chosen because it provided stable, interpretable, and well-calibrated performance across folds. Ridge regularization further mitigated overfitting by shrinking coefficients in the correlated, high-dimensional setting. The final selected logistic regression model was then trained using hyperparameter optimization and five-fold cross-validation, optimized for AUROC.

The finalized model consisted of 29 genes—13 downregulated and 16 upregulated in association with early acute rejection. Scores were reported on a standardized scale from 0-100, with higher

values reflecting increased risk of EAR. This final model was locked and implemented in the prediction pipeline for validation. The full list of the 29 genes, including name, Ensembl ID, functional roles, and supporting references, is provided in **Supplemental Table 1**.

2. Supplemental Figure 1. Identification of differentially expressed genes in pre-transplant blood

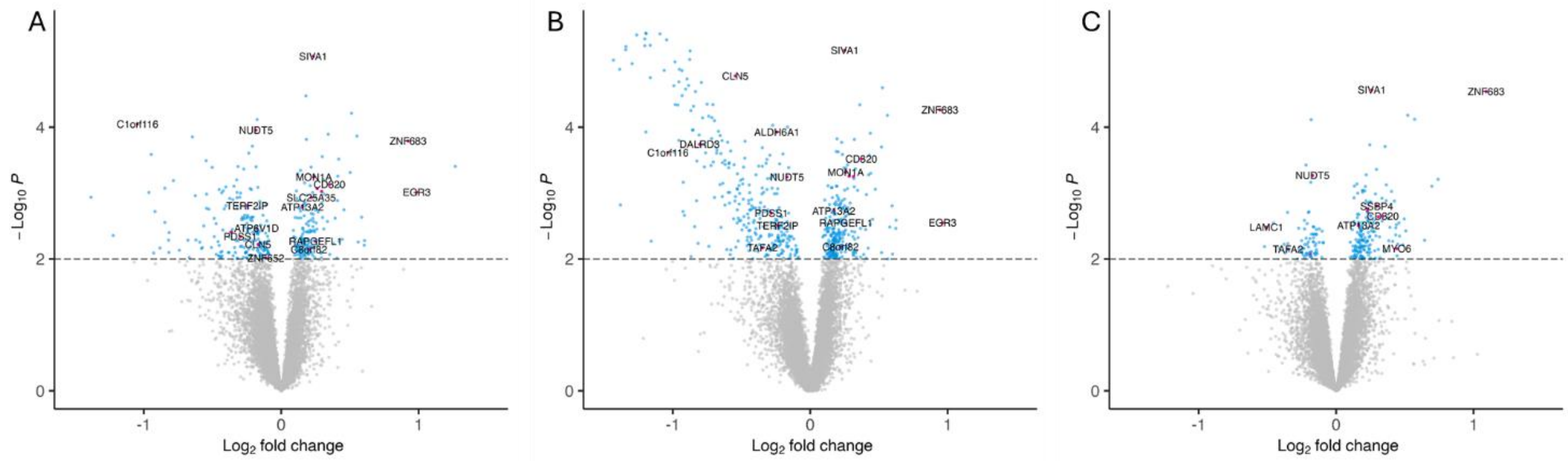

Volcano plots generated using three differential expression analysis methods to compare pre-transplant blood samples from patients (n=123) who developed early acute rejection post-transplant and those who did not. (A) DESeq2; (B) EdgeR; and (C) Limma. Genes included in the final 29-gene PTRA signature are highlighted in blue and labeled.

3. Supplemental Table 1: List of 29 genes included in PRTA signature assay

Gene Ensembl ID/ Name	Known or Postulated Role(s)	Relevant References
**ENSG00000119711 ALDH6A1** Aldehyde Dehydrogenase 6 Family Member A1	ALDH6A1 encodes a member of the aldehyde dehydrogenase protein family and is involved in immune system modulation and NADPH synthesis.[1] Gene expression level of ALDH6A1 was found to suppress the growth and migration of renal clear cell carcinoma.[35-36]	Xia et al. [1] Protein Atlas[35] Stenvinkel et. al.[36]
**ENSG00000159363 ATP13A2** ATPase Cation Transporting 13A2	ATP13A2 has been directly linked to mitophagy and plays an important role in maintaining cellular health by regulating lysosomal function, autophagy, and mitochondrial homeostasis.[2] ATP13A2 dysregulation can lead to vascular inflammation, increased permeability, and endothelial stress, all of which may contribute to immune-mediated damage.[37] Gene expression level of ATP13A2 is also associated with favorable outcomes in renal cancer.[35]	Lopes da Fonseca et al. [2] Protein Atlas[35] Tsunemi et. al.[37]
**ENSG00000100554 ATP6V1D** ATPase H+ Transporting V1 Subunit D	V-ATPase is a multisubunit enzyme that hydrolyzes ATP and translocates protons across membranes. V-ATPase is responsible for acidifying and maintaining the pH of intracellular compartments.[3] V-ATPase is involved in the activation of innate immune cells by regulating pH levels and the trafficking of proteins necessary for immune response.[38] One study found that there is a strong association between ATP6V1D dysregulation and clear cell renal carcinoma, suggesting that ATP6V1D offers a protective role in disease progression in the kidney.[4] Gene expression level of ATP6V1D is also associated with favorable outcomes in renal cancer.[35]	Wang et al.[3] Li et al.[4] Protein Atlas[35] Sun-Wang[38]
**ENSG00000123838 C4BPA** Complement component 4 binding protein alpha	Along with a single, unique beta-chain, seven identical alpha-chains encoded by C4BPA assemble into the predominant isoform of C4b-binding protein (C4BP), a multimeric protein that controls activation of the complement cascade through the classical pathway.[5] In addition to being involved in innate immune response and T cell-mediated immunity in gene ontology terms, subunit C4d of the C4BP structure has been reported to be an important tool for the diagnosis of alloantibody-dependent allograft injury independent from its occurrence early or late after transplantation.[6] Gene expression level of C4BPA is associated with unfavorable outcomes in renal cancer.[35]	Merle et al.[5] Watschinger et al.[6] Protein Atlas[35]
**ENSG00000179388 EGR3** Early Growth Response 3	EGR3 is a transcription factor belonging to the EGR family of C2H2-type zinc-finger proteins. It functions as an immediate-early growth response gene induced by mitogenic stimulation and is essential for the control of inflammation and antigen-induced proliferation of B and T cells. This gene regulates B and T cell function in adaptive immune responses and homeostasis by promoting antigen receptor signaling and controlling inflammation.[7] Deficiencies in EGR3 have been linked to altered T cell responses.[7] EGR3 regulates B cell receptor (BCR) signaling and is involved in B cell activation and differentiation, which are critical for donor-specific antibody (DSA) production in transplant rejection.[39] EGR3 modulates cytokine production in T cells, macrophages, and dendritic cells, influencing inflammatory responses in both innate and adaptive immunity.[40]	Li et al.[7] Zhou et.al.[39] Morita et.al.[40]
**ENSG00000135862 LAMC1** Laminin Subunit Gamma 1	Laminins are a family of extracellular matrix (ECM) glycoproteins, which are implicated in a wide variety of biological processes including cell adhesion, differentiation, migration, and signaling. Laminins are also main glycoproteins in the composition of glomerular basement membranes. Gene Set Enrichment Analysis (GSEA) revealed that the LAMC1 high expression phenotype is associated with adherens junctions, ECM receptor interaction, the MAPK signaling pathway, and the TGF-β signaling pathway.[8] Gene expression level of LAMC1 is associated with favorable outcomes in renal cancer.[35]	Bai et al.[8] Protein Atlas[35]

**ENSG00000164077 MON1A** MON1 homolog A, secretory trafficking associated	MON1A enables guanyl-nucleotide exchange factor activity and is predicted to be involved in protein secretion, acting upstream of or within cellular homeostasis and protein transport.[9] MONA1A plays a significant role in iron metabolism by facilitating the trafficking of ferroportin, the primary iron exporter, to the surface of iron-recycling macrophages.[41] Gene expression level of MON1A is associated with unfavorable outcomes in renal cancer.[35]	Raudenska et al.[9] Protein Atlas[35] Wang et.al.[41]
**ENSG00000178802 MPI** Mannose Phosphate Isomerase	MPI plays a critical role in glycosylation reactions. In a mouse model, deficiency in glycosylation has been linked to reduced intercellular adhesion molecule-1 (ICAM-1), which in turn impacts the innate immune response and leads to sustained inflammatory response and tissue damage.[10] Gene expression level of MPI is associated with favorable outcomes in renal cancer.[35]	He et. al.[10] Protein Atlas[35]
**ENSG00000196586 MYO6** Myosin VI	The actin motor protein MYO6 is a multivalent protein with diverse functions, playing critical roles in intracellular transport, endocytosis, and vesicular trafficking. Mutations of MYO6 can cause polycystic kidney disease.[11] MYO6 shows potential as both a diagnostic and prognostic marker in ccRCC and is associated with tumor-infiltrating immune cells, particularly macrophages. MYO6 expression is significantly lower in ccRCC tumors compared to normal tissues, with lower levels linked to higher cancer grade and later TNM stage.[42] Gene expression level of MYO6 is associated with favorable outcomes in renal and urothelial cancers.[35]	Magistrati et al.[11] Protein Atlas[35] Zhang et.al.[42]
**ENSG00000099795 NDUFB7** NADH:ubiquinone oxidoreductase subunit B7	NDUFB7 is involved in mitochondrial complex I, which is the primary entry point for electrons into the electron transport chain, required for the bulk of cellular ATP production via oxidative phosphorylation.[12] Playing an important role in energy production, NDUFB7 was identified to be associated with the proliferation pathways of kidney disease development– increased proliferation and decreased mitochondrial function was associated with poor renal outcomes. Proliferation is an energy-demanding process impairing the cellular ability to cope with a toxic or ischemic injury, identifying the association of proliferative repair and metabolic recovery as indispensable and interdependent features for successful kidney repair.[13]	Formosa L et al.[12] Galichon P et al.[13]
**ENSG00000165609 NUDT5** Nudix hydrolase 5	NUDT5 is involved in the ribonucleotide metabolic process pathway and discriminates patients developing delayed graft function (DGF) from those having early graft function (EGF) at the time of transplantation.[14] Gene expression level of NUDT5 is associated with unfavorable outcomes in renal cancer.[35]	Zaza et al.[14] Protein Atlas[35]
**ENSG00000157911 PEX10** Peroxisomal Biogenesis Factor 10	PEX10 encodes a protein involved in the import of peroxisomal matrix proteins and interactions with other PEX proteins, such as PEX5 and PEX 19. The kidney has a high density of peroxisomes, which actively participate in apoptosis, innate immunity, and inflammation, and in the pathogenesis of kidney injury.[15] Gene expression level of PEX10 is associated with favorable outcomes in renal cancer.[35]	Vasko R.[15] Protein Atlas[35]
**ENSG00000184990 SIVA1** SIVA1 Apoptosis-Inducing Factor	SIVA1 encodes a pro-apoptotic E3 ubiquitin ligase that regulates cell cycle progression, cell proliferation, and apoptosis.[16] SIVA1 modulates cytokine production by influencing the NF-κB pathway, which regulates pro-inflammatory signaling. It also interacts with CD27, a TNFR family member, affecting T cell activation-induced cell death and cytokine regulation.[43-44]	Genecards[16] Resch et. al.[43] Py et.al.[44]
**ENSG00000198740 ZNF652** Zinc Finger Protein 652	ZNF652 has been reported to play a transcription repressor role in regulating the TGF-b pathway and natural killer cells in various cancers.[17,18] In the context of kidney injury, ZNF652 has been shown to influence the expression of genes involved in inflammation and immune responses, which are central to cytokine and chemokine production.[45] Gene expression level of ZNF652 is associated with favorable outcomes in renal cancer.[35]	Madrigal T et al.[17] Ke et al.[18] Protein Atlas[35] Kesarwani et.al.[45]

**ENSG00000176083**<br>**ZNF683**<br>Zinc Finger Protein 683	Analysis of renal allograft biopsies reveals that ZNF683 was indicated in the memory $CD8^+$ T cell phenotype.[19] Further, the ZNF683/Hobit transcription factor contributes to early-stage development of NK cells, promotes ILC1 effector differentiation, reduces cytolytic activity, and reduces IFNγ production.[20] ZNF683 is a critical regulator of cytokine and chemokine production, especially in Th2 cells. It promotes the production of IL-4, IL-5, and IL-13, cytokines associated with immune responses and immune cell recruitment critical in the regulation of T cell responses.[46] Gene expression level of ZNF683 is associated with favorable outcomes in urothelial cancer.[35]	Shi et al.[19]<br>Zingoni et al.[20]<br>Protein Atlas[35]<br>Post et. al.[46]
**ENSG00000125434**<br>**SLC25A35**<br>Solute carrier family 25, member 35	SLC25A35 belongs to the SLC25 family of mitochondrial carrier proteins. SLC25A35 plays a critical role in maintaining mitochondrial function by facilitating the exchange of metabolites across the inner mitochondrial membrane.[47] Gene expression level of SLC25A35 is associated with favorable outcomes in renal and urothelial cancers.[35]	Protein Atlas[35]<br>Yu et.al.[47]
**ENSG00000105072**<br>**C19orf44**<br>Chromosome 19 open reading frame 44	C19orf44 is a relatively understudied protein encoding gene, but it has been implicated in various biological processes, including variations in lymphocyte counts and immune response,[48] cancer biology, and cellular signaling pathways.	Nalls et.al.[48]
**ENSG00000182795**<br>**C1orf116**<br>Chromosome 1 open reading frame 116	C1orf116 is a novel driver of the epithelial phenotype. In particular, C1orf116 expression promotes cell-cell tight junctions.[21,22] Gene expression level of C1orf116 is associated with favorable outcomes in renal cancer.[35]	Amend et al.[21]<br>Amend et al.[22]<br>Protein Atlas[35]
**ENSG00000213563**<br>**C8orf82**<br>Chromosome 8 Open Reading Frame 82	C8orf82 encodes a protein whose function remains largely unclear. Initial studies suggest C8orf82 is involved in the regulation of immune responses, with altered expression levels observed in cancer and inflammatory diseases.[49] Gene expression level of C8orf82 is associated with favorable outcomes in renal cancer.[35]	Li et.al.[49]<br>Protein Atlas[35]
**ENSG00000167775**<br>**CD320**<br>CD320 Molecule	CD320 facilitates NK cell activation[23] and is associated with gene ontology terms of positive regulation of B cell proliferation and B cell co-stimulation. CD320's engagement on immune cells enhances the production of pro-inflammatory cytokines like TNF-α and IL-6, which are critical for recruiting additional immune cells to sites of infection or injury.[50]	Shilts et al.[23]<br>Liu et.al.[50]
**ENSG00000102805**<br>**CLN5**<br>CLN5 intracellular trafficking protein	CLN5 is involved in the activation of mitophagy and mitochondrial homeostasis. One study demonstrated that loss of CLN5 leads to dysregulation of mitochondrial proteins, resulting in oxidative stress, bioenergetic impairment, and activation of autophagy pathways that ultimately trigger mitophagy.[24] Gene expression level of CLN5 is associated with favorable outcomes in renal cancer.[35]	Doccini et al.[24]<br>Protein Atlas[35]
**ENSG00000178149**<br>**DALRD3**<br>DALR Anticodon Binding Domain Containing 3	DALRD3 is a poorly characterized gene that encodes a protein involved in tRNA methylation that specifically modifies arginine tRNAs. While its exact biological function remains unclear, it has been implicated in neurological development and disease.[51] Gene expression level of DALRD3 is associated with favorable outcomes in renal cancer.[35]	Lentini et.al.[51]<br>Protein Atlas[35]
**ENSG00000176845**<br>**METRNL**<br>Meteorin like, glial cell differentiation regulator	METRNL is a cytokine involved in various immune responses and cellular functions. METRNL regulates lipid metabolism in the kidney by modulating mitochondrial function and is a stress-responsive regulator of kidney pathophysiology.[25] Overexpression of METRNL inhibits adipose inflammation, enhances adipocyte differentiation, activates lipid metabolism, and plays a neuroprotective role.[26]	Zhou et al.[25]<br>Zheng et al.[26]
**ENSG00000148459**<br>**PDSS1**<br>Decaprenyl diphosphate synthase, subunit 1	PDSS1 elongates the prenyl side chain of coenzyme Q in the quinone biosynthesis pathway, which results in a series of coenzyme Q (important antioxidants in the cell's ability to combat oxidative stress).[27] Dysfunction in coenzyme Q synthesis pathways can lead to renal dysfunction and	Mollet et al.[27]<br>Berardo et al.[28]<br>Protein Atlas[35]

	tubulopathy.[28] Gene expression level of PDSS1 is associated with favorable outcomes in renal cancer.[35]	
**ENSG00000108352**<br>**RAPGEFL1**<br>Rap guanine nucleotide exchange factor like 1	RAPGEFL1 is not well characterized but is predicted to enable guanyl-nucleotide exchange factor activity. Gene expression level of RAPGEFL1 is associated with unfavorable outcomes in renal cancer.[35]	Protein Atlas[35]
**ENSG00000185187**<br>**SIGIRR**<br>Single Ig And TIR Domain Containing	TIR8/SIGIRR inhibits ILR signaling, TLR signaling, and NF-κB activation.[29] Reported gene ontology terms associated with SIGIRR are negative regulation of cytokine-regulated signaling pathway, acute-phase response, and negative regulation of chemokine production. Through its regulatory effects on TLRs and IL-1Rs, SIGIRR affects the recruitment and activation of innate immune cells.[52] In models of renal ischemia/reperfusion or kidney transplantation, Tir8/Sigirr-deficient mice showed increased renal injury or severe graft rejection, respectively, associated with excessive cytokine and chemokine production and consequently, leukocyte recruitment and amplified adaptive immune response against donor antigens.[30] Gene expression level of SIGIRR is associated with favorable outcomes in renal cancer.[35]	Riva et al.[29]<br>Garlanda et al.[30]<br>Protein Atlas[35]<br>Xu et.al.[52]
**ENSG00000130511**<br>**SSBP4**<br>Single Stranded DNA Binding Protein 4	SSBP4 is a human gene encoding the single-stranded DNA-binding protein 4, which is predicted to enable single-stranded DNA binding activity and to be involved in the positive regulation of transcription by RNA polymerase II. Gene expression level of SSBP4 is associated with unfavorable outcomes in renal cancer.[35]	Protein Atlas[35]
**ENSG00000198673**<br>**TAFA2**<br>TAFA chemokine like family member 2	TAFA2 is a member of the TAFA chemokine-like ligand or FAM19A family, which is involved in the regulation of a diverse range of cellular processes, including inflammation, tissue fibrosis, chemotaxis of macrophages, phagocytosis, and release of reactive oxygen species (ROS).[31] Further, TAFA2 directly binds to the lectin-like domain of ADGRL1, activating the cAMP/PKA/CREB/BCL2 signaling pathway, which is crucial in preventing cell death.[32]	Zhu et al.[31]<br>Liang et al.[32]
**ENSG00000166848**<br>**TERF21P**<br>TERF2 interacting protein	TERF2IP acts as a regulator of telomere function and as a transcriptional regulator. In hyperglycemia, expression is inhibited leading to tubular cell injury associated with diabetic nephropathy. When cytoplasmic, it associates with the I-kappa-B-kinase (IKK) complex and activates expression of NF-kB target genes.[33] TERF21P has also been reported to reduce renal tubular injury in diabetic nephropathy.[34] Gene expression level of TERF21P is associated with favorable outcomes in renal cancer.[35]	Atlasgenetics[33]<br>Deregowska et al.[34]<br>Protein Atlas[35]

Supplemental Figure 2: ROC Curve for Class I and Class II PRA

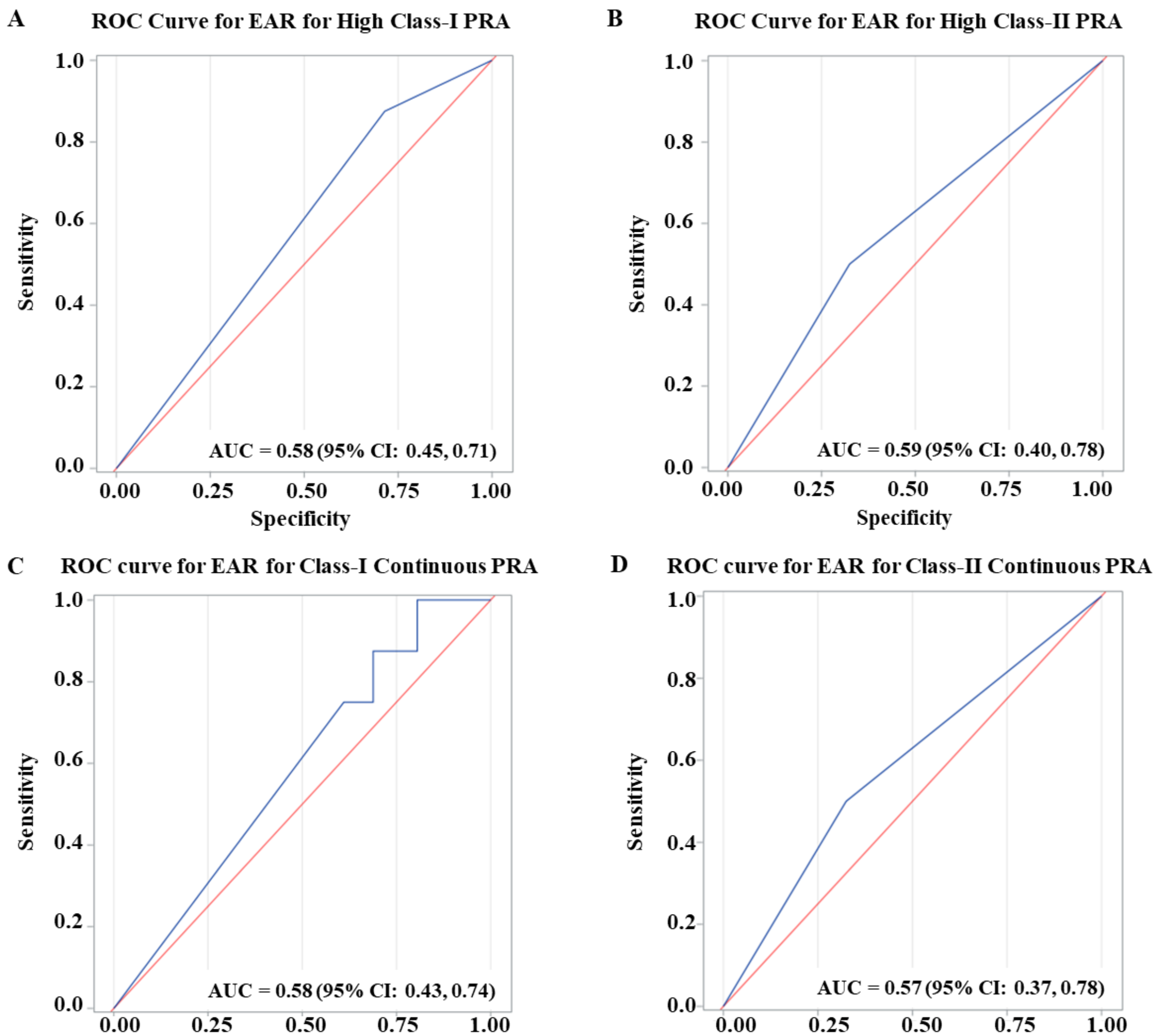

ROC for Class I and Class II PRA levels for comparison treated as a dichotomous (A and B) and as a continuous risk (C and D).

AUC, area under the curve; EAR, early acute rejection; PRA, panel reactive antibodies; ROC, receiver operating characteristic

References

1. Xia G, Wu S, Cui X, et al. An immune infiltration-related prognostic model of kidney renal clear cell carcinoma with two valuable markers: CAPN12 and MSC. Sec. *Front. Oncol.* 2023; 13:1161666. doi: 10.3389/fonc.2023.1161666.
2. Lopes da Fonseca T, Outeiro T, et al. ATP13A2 and Alpha-synuclein: a Metal Taste in Autophagy. *Exp Neurobiol.* 2014;23(4):314-23. doi: 10.5607/en.2014.23.4.314.
3. Wang L, Wu D, Robinson C, et al. Structures of a Complete Human V-ATPase Reveal Mechanisms of Its Assembly. *Mol Cell.* 2020;80(3):501-511.e3. doi: 10.1016/j.molcel.2020.09.029.
4. Li X, Li H, Yang C, et al. Comprehensive Analysis of ATP6V1s Family Members in Renal Clear Cell Carcinoma With Prognostic Values. *Front Oncol.* 2020;10:567970. doi: 10.3389/fonc.2020.567970.
5. Merle N, Church S, Fremeaux-Bacchi V, et al. Complement System Part I - Molecular Mechanisms of Activation and Regulation. *Front Immunol*. 2015;6:262. doi: 10.3389/fimmu.2015.00262.
6. Watschinger B, Pascual M, et al. Capillary C4d deposition as a marker of humoral immunity in renal allograft rejection. *J Am Soc Nephrol.* 2002;13(9):2420-3. doi: 10.1097/01.asn.0000029941.34837.22.
7. Li S, Miao T, Sebastian M et al. The transcription factors Egr2 and Egr3 are essential for the control of inflammation and antigen-induced proliferation of B and T cells. *Immunity*. 2012;37(4):685-96. doi: 10.1016/j.immuni.2012.08.001.

8. Bai J, Zheng A, Ha Y, et al. Comprehensive analysis of LAMC1 expression and prognostic value in kidney renal papillary cell carcinoma and clear cell carcinoma. *Front Mol Biosci*. 2022:9:988777. doi: 10.3389/fmolb.2022.988777.
9. Raudenska M, Balvan J, Masarik M, et al. Crosstalk between autophagy inhibitors and endosome-related secretory pathways: a challenge for autophagy-based treatment of solid cancers. *Mol Cancer*. 2021;20(1):140. doi: 10.1186/s12943-021-01423-6.
10. He P, Srikrishna G, Freeze H, et al. N-glycosylation deficiency reduces ICAM-1 induction and impairs inflammatory response. *Glycobiology*. 2014;24(4):392-8. doi: 10.1093/glycob/cwu006.
11. Magistrati E, Maestrini G, Niño C. et al. Myosin VI regulates ciliogenesis by promoting the turnover of the centrosomal/satellite protein OFD1. *EMBO Rep*. 2022;23(3):e54160. doi: 10.15252/embr.202154160.
12. Formosa L, Dibley M, Stroud D, et al. Building a complex complex: Assembly of mitochondrial respiratory chain complex I. *Semin Cell Dev Biol*. 2018:76:154-162. doi: 10.1016/j.semcdb.2017.08.011.
13. Galichon P, Lannoy M, Li L, et al. Kidney epithelial proliferation impairs cell viability via energy depletion. *bioRxiv*. 2020;20:49. doi.org/10.1101/2020.04.24.054643.
14. Zaza G, Rascio F, Pontrelli P, et al. Karyopherins: potential biological elements involved in the delayed graft function in renal transplant recipients. *BMC Med Genomics*. 2014:7:14. doi: 10.1186/1755-8794-7-14.
15. Vasko R. Peroxisomes and Kidney Injury. *Antioxid Redox Signal*. 2016;25(4):217-31. doi: 10.1089/ars.2016.6666.

16. GeneCards https://www.genecards.org/cgi-bin/carddisp.pl?gene=SIVA1&keywords=ENSG00000184990

17. Madrigal T, Hernández-Monge J, Herrera L, et al. Regulation of miRNAs Expression by Mutant p53 Gain of Function in Cancer. *Front Cell Dev Biol*. 2021;9:695723. doi: 10.3389/fcell.2021.695723.

18. Ke H, Zhang J, Wang F, et al. ZNF652-Induced circRHOT1 Promotes SMAD5 Expression to Modulate Tumorigenic Properties and Nature Killer Cell-Mediated Toxicity in Bladder Cancer via Targeting miR-3666. *J Immunol Res*. 2021;2021:7608178. doi: 10.1155/2021/7608178.

19. Shi T, Burg A, Caldwell J, et al. Single-cell transcriptomic analysis of renal allograft rejection reveals insights into intragraft TCR clonality. *J Clin Invest.* 2023;133(14):e170191. doi: 10.1172/JCI170191.

20. Zingoni A, Cippitelli M. Rescue SLAMF7 activity in exhausted natural killer cells: Novel challenges in the immunotherapy of multiple myeloma. *Clin. Transl. Disc.* 2022:2e143. https://doi.org/10.1002/ctd2.143.

21. Amend S, Hernandez J, Parsana P, et al. Abstract 853: C1orf116, a gene with unknown function, is a novel driver of epithelial phenotype in epithelial-to-mesenchymal transition in human cancer. *Cancer Res*. 2017;77 (13_Supplement): 853. https://doi.org/10.1158/1538-7445.AM2017-853

22. Amend S, Pienta K. Abstract 3156: C1orf116, an uncharacterized gene with unknown function, restricts movement capacity of would-be metastatic cells through modulation of tight junctions *Cancer Res* (2018) 78 13_Supplement): 3156. https://doi.org/10.1158/1538-7445.AM2018-3156

23. Shilts J, Severin Y, Galaway F, et al. A physical wiring diagram for the human immune system. *Nature*. 2022;608, 397–404. https://doi.org/10.1038/s41586-022-05028-x.

24. Doccini S, Morani F, Nesti C, et al. Proteomic and functional analyses in disease models reveal CLN5 protein involvement in mitochondrial dysfunction. *Cell Death Discov*. 2020;6,18. https://doi.org/10.1038/s41420-020-0250-y.

25. Zhou Y, Liu L, Jun B, et al. Metrnl Alleviates Lipid Accumulation by Modulating Mitochondrial Homeostasis in Diabetic Nephropathy. *Diabetes*. 2023;72(5):611-626. doi: 10.2337/db22-0680.

26. Zheng S, Li Z, Song J, et al. Metrnl: a secreted protein with new emerging functions. *Acta Pharmacol Sin.* 2016;37(5):571-9. doi: 10.1038/aps.2016.9.

27. Mollet J, Giurgea I, Schlemmer D, et al. Prenyldiphosphate synthase, subunit 1 (PDSS1) and OH-benzoate polyprenyltransferase (COQ2) mutations in ubiquinone deficiency and oxidative phosphorylation disorders. *J Clin Invest*. 2007;117(3):765-72. doi: 10.1172/JCI29089.

28. Berardo A, Quinzii C. Redefining infantile-onset multisystem phenotypes of coenzyme Q10-deficiency in the next-generation sequencing era. *J Transl Genet Genom*. 2020:4:22-35. doi: 10.20517/jtgg.2020.02.

29. Riva F, Bonavita E, Barbati E, et al. TIR8/SIGIRR is an interleukin-1 receptor/toll like receptor family member with regulatory functions in inflammation and immunity. *Front. Immunol*. 2012;3:322. https://doi.org/10.3389/fimmu.2012.00322.

30. Garlanda C, Riva F, Bonavita E, et al. Decoys and Regulatory "Receptors" of the IL-1/Toll-Like Receptor Superfamily. *Front Immunol*. 2013;4:180. doi: 10.3389/fimmu.2013.00180.

31. Zhu S, Hu X, Bennett S, et al. Molecular Structure, Expression and Role of TAFA4 and its Receptor FPR1 in the Spinal Cord. *Front Cell Dev Biol*. 2022;10:911414. doi: 10.3389/fcell.2022.911414.
32. Liang H, Tang L, Ge H,et al. Neuronal survival factor TAFA2 suppresses apoptosis through binding to ADGRL1 and activating cAMP/PKA/CREB/BCL2 signaling pathway. *Life Sciences*. 2023;334:122241. https://doi.org/10.1016/j.lfs.2023.122241.
33. www.atlasgeneticsoncology.org/Genes/SIVA1ID42301ch14q32.html
34. Deregowska A, Wnuk M. RAP1/TERF2IP—A Multifunctional Player in Cancer Development. *Cancers.* 2021;13(23), 5970; https://doi.org/10.3390/cancers13235970.
35. Protein Atlas https://www.proteinatlas.org/
36. Stenvinkel P, Zoccali C, Ikizler TA. Energy metabolism and wasting in chronic kidney disease. *Kidney Int*. 2013;84(3):623-627. doi:10.1038/ki.2013.147.
37. Tsunemi T, Hamada K, Krainc D. ATP13A2/PARK9 regulates secretion of exosomes and α-synuclein. *J Neurosci*. 2019
38. Sun-Wang JL, Ivanova S, Zorzano A. The role of V-ATPase in immune cell function. *Front Immunol*. 2021
39. Zhou Y, Zhang J, Lu Q, et al. Egr3, a critical regulator of B cell function and autoimmunity. *Nat Immunol*. 2008
40. Morita K, Okamura T, Inoue M, et al. Egr3 regulates pro-inflammatory cytokine production and effector function of T cells. *J Immunol*. 2016
41. Wang F, Paradkar PN, Custodio AO, et al. Genetic variation in Mon1a affects protein trafficking and modifies macrophage iron loading in mice. *Nat Genet*. 2007;39(8):1025-1032. doi:10.1038/ng2059.

42. Zhang Y, Li Y, Wang L, et al. A comprehensive analysis of MYO6 as a promising biomarker for diagnosis, prognosis, and immunity in clear cell renal cell carcinoma. *Front Oncol*. 2021;11:698346. doi:10.3389/fonc.2021.698346.

43. Resch U, Schichl YM, Winsauer G, Gudi R, Prasad K, de Martin R. Siva1 is a XIAP-interacting protein that balances NFκB and JNK signalling to promote apoptosis. *J Cell Sci*. 2009;122(Pt 15):2651-2661. doi:10.1242/jcs.049940.

44. Py B, Slomianny C, Auberger P, Petit PX, Benichou S. Siva-1 and an alternative splice form lacking the death domain, Siva-2, similarly induce apoptosis in T lymphocytes via a caspase-dependent mitochondrial pathway. *J Immunol*. 2004;172(7):4008-4017. doi:10.4049/jimmunol.172.7.4008.

45. Kesarwani A, Gupta R, Yadav A, et al. Role of transcription factors in inflammation-driven kidney injury. *J Immunol Res*. 2021.

46. Post M, Cuapio A, Osl M, et. al. The Transcription Factor ZNF683/HOBIT Regulates Human NK-Cell Development. *Front Immunol*. 2017 May 15;8:535.

47. Yu H, Bai L, Jin L, et. al. SLC25A35 enhances fatty acid oxidation and mitochondrial biogenesis to promote the carcinogenesis and progression of hepatocellular carcinoma by upregulating PGC-1α. *Cell Communication and Signaling* (2025) 23:130 https://doi.org/10.1186/s12964-025-02109-y

48. Nalls MA, Couper DJ, Tanaka T, et.al. Multiple loci are associated with white blood cell phenotypes. *PLoS Genet*. 2011 Jun;7(6):e1002113. doi: 10.1371/journal.pgen.1002113.

49. Li J, Wang X, Zhao Y, et al. C8orf82 as a novel biomarker for cancer and inflammation-related disorders. *Front Immunol.* 2021;12:652013.

50. Liu Q, Zhang H, Li Y, et al. Role of CD320 in monocyte/macrophage activation in immune responses. *Front Immunol*. 2019;10:347. doi:10.3389/fimmu.2019.00347.

51. Lentini JM, Alsaif HS, Faqeih E, Alkuraya FS, Fu D. DALRD3 encodes a protein mutated in epileptic encephalopathy that targets arginine tRNAs for 3-methylcytosine modification. *Nat Commun*. 2020;11(1):2510. doi:10.1038/s41467-020-16321-6.

52. Xu Y, Tao X, Shen B, et al. Regulation of CD18 stability by SIGIRR-modulated ubiquitination: new insights into the relationship between innate immune response and acute lung injury. *J Immunol*. 2010;184(7):4021-4029. doi:10.4049/jimmunol.0903500.
